# Dynamics of rice microbiomes reveal core vertically transmitted seed endophytes

**DOI:** 10.1186/s40168-022-01422-9

**Published:** 2022-12-09

**Authors:** Xiaoxia Zhang, Yi-Nan Ma, Xing Wang, Kaiji Liao, Shanwen He, Xia Zhao, Hebao Guo, Dongfang Zhao, Hai-Lei Wei

**Affiliations:** Key Laboratory of Microbial Resources Collection and Preservation, Ministry of Agriculture and Rural Affairs, Institute of Agricultural Resources and Regional Planning, Chinese Academy of Agricultural Sciences, Beijing, 100081 China

**Keywords:** High-throughput sequencing, Rice microbiome, Seed endophytic microbiota, Vertical transmission, Plant growth promotion

## Abstract

**Background:**

Plants and their associated microbiota constitute an assemblage of species known as holobionts. The plant seed microbiome plays an important role in nutrient uptake and stress attenuation. However, the core vertically transmitted endophytes remain largely unexplored.

**Results:**

To gain valuable insights into the vertical transmission of rice seed core endophytes, we conducted a large-scale analysis of the microbiomes of two generations of six different rice varieties from five microhabitats (bulk soil, rhizosphere, root, stem, and seed) from four geographic locations. We showed that the microhabitat rather than the geographic location and rice variety was the primary driver of the rice microbiome assemblage. The diversity and network complexity of the rice-associated microbiome decreased steadily from far to near the roots, rice exterior to interior, and from belowground to aboveground niches. Remarkably, the microbiomes of the roots, stems, and seeds of the rice interior compartments were not greatly influenced by the external environment. The core bacterial endophytes of rice were primarily comprised of 14 amplicon sequence variants (ASVs), 10 of which, especially ASV_2 (*Pantoea*) and ASV_48 (*Xanthomonas*), were identified as potentially vertically transmitted taxa because they existed across generations, were rarely present in exterior rice microhabitats, and were frequently isolated from rice seeds. The genome sequences of *Pantoea* and *Xanthomonas* isolated from the parental and offspring seeds showed a high degree of average nucleotide and core protein identity, indicating vertical transmission of seed endophytes across generations. In silico prediction indicated that the seed endophytes *Pantoea* and *Xanthomonas* possessed streamlined genomes with short lengths, low-complexity metabolism, and various plant growth-promoting traits. We also found that all strains of *Pantoea* and *Xanthomonas* exhibited cellulase activity and produced indole-3-acetic acid. However, most strains exhibited insignificant antagonism to the major pathogens of rice, such as *Magnaporthe oryzae* and *X. oryzae* pv. *oryzae*.

**Conclusion:**

Overall, our study revealed that microhabitats, rather than site-specific environmental factors or host varieties, shape the rice microbiome. We discovered the vertically transmitted profiles and keystone taxa of the rice microbiome, which led to the isolation of culturable seed endophytes and investigation of their potential roles in plant-microbiome interactions. Our results provide insights on vertically transmitted microbiota and suggest new avenues for improving plant fitness via the manipulation of seed-associated microbiomes.

Video Abstract

**Supplementary Information:**

The online version contains supplementary material available at 10.1186/s40168-022-01422-9.

## Background

Microorganisms are present in all stages of plant development. Healthy plant growth is dependent on homeostasis, which is largely maintained by three factors: environment, host genetics, and the microbiome [1]. Changes in stability may result in the emergence of plant diseases or maldevelopment. Plants and their associated microbiota act as a functional entity, known as the “holobiont” [2, 3]. Plant microbiota can be extracellular, intracellular, acquired from the environment, or inherited from the parent, which is called vertical transmission [4, 5]. The shaping of plant-associated microbiota is not stochastic but is determined by soil type, geographic location, host genotype, and plant compartments. All plant compartments can serve as microhabitats for diverse microorganisms to regulate holobiont fitness and resistance to environmental changes [2].

Seed, as a reproductive organ, represents an exclusive niche for a unique microbiota that harbors various adaptations for successful colonization [6]. Seed-associated microbes play roles in nutrient intake and reduction of abiotic and biotic stress [7]. Bacterial isolates from plant seeds can solubilize phosphorus, fix nitrogen, produce growth hormones, and synthesize antimicrobial compounds [8, 9]. The conserved beneficial features of seed microbiota reflect the possible requirement of host seeds for a protective layer [6]. Seed endophytes are of particular interest among seed-associated microbes because some can accompany plants throughout their life cycle, from germination to development, growth, and fruiting [7, 10, 11] as well as during postharvest storage and processing [12–14]. Particularly, seed core endophytes coevolve with their hosts as a “continuity of partnership” and develop a robust and efficient transmission strategy over generations [11, 15, 16]. Therefore, in-depth analysis of the composition of the seed core endophytic microbiota will be useful not only for systematically understanding the coevolutionary mechanism of holobionts but also for the mining, development, and utilization of functional microbial resources.

Rice (*Oryza sativa*) is a model plant and a staple crop worldwide. Several pioneering studies have attempted to characterize the taxonomic profiles of rice-associated microbiota, define the core microbiota of the rice rhizosphere and seed, and infer the driving factors shaping the rice microbial community [17–22]. However, there is limited data on the changes in seed endophytic microbiome different compartments of rice. Little is known about species- or strain-level transmission patterns across generations. To address these issues and gain valuable insights into the vertical transmission of rice seed core endophytes, we conducted a large-scale analysis of the microbiomes of two generations of six different rice varieties from five microhabitats (bulk soil, rhizosphere, root, stem, and seed) at four geographic locations. Our findings revealed that 14 amplicon sequence variants (ASVs) distributed in 12 known genera and one unclassified genus coexisted throughout the life cycle of rice, regardless of rice variety, microhabitat, or geographic location. Among the core microbial groups, 10 ASVs, including ASV_2 of *Pantoea* with three species and ASV_48 of *Xanthomonas* with one species, were the most abundant vertically transmittable bacteria within the seed endotypes. Moreover, the genome sequences of *Pantoea* and *Xanthomonas* isolated from the parental seed and offspring seeds showed a high degree of average nucleotide identity and core protein identity, indicating the vertical transmission of seed endophytes across generations. Additionally, we determined the plant growth-promoting and plant protection-associated features of the isolates via in silico prediction and experimental tests.

## Results

### Quality metrics of sequencing analysis

**Fig. 1:**
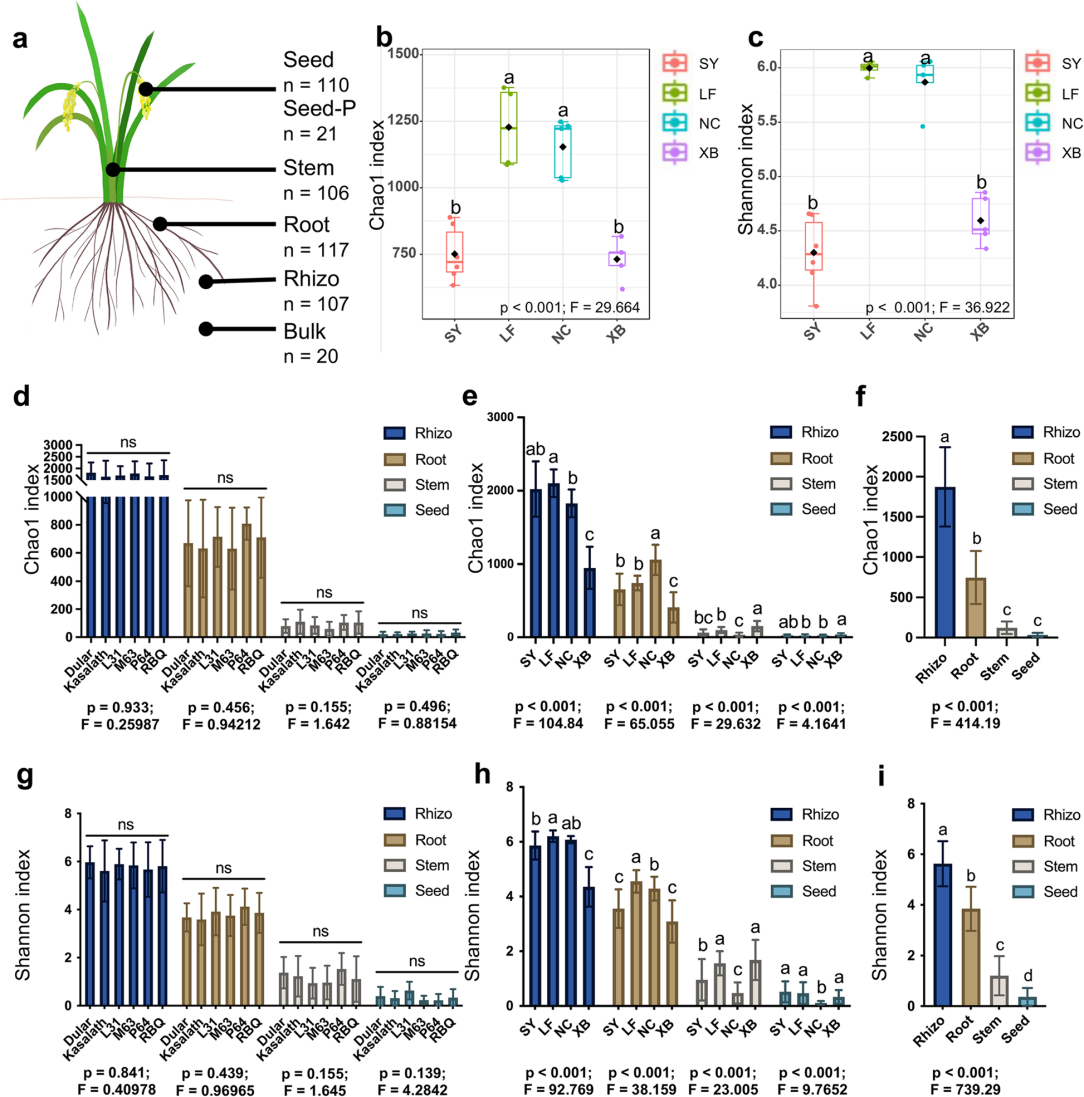
Alpha diversity of rice-associated microbiomes. **a** Samples were collected from five microhabitats. Bulk, bulk soil; rhizo, rhizosphere; root, root endosphere; stem, stem endosphere; seed, offspring seed endosphere; seed-P, parental seed endosphere. n represents the sample numbers. **b** and **c** Chao1 and Shannon index of the microbiota of bulk soil from four regions. SY, Sanya; LF, Langfang; NC, Nanchang; XB, Xishuangbanna. Horizontal bars within boxes represent medians. Tops and bottoms of boxes represent the 75th and 25th percentiles, respectively. Statistical significance was determined using ANOVA and *T*-test. Lower letters represent the statistical differences at the 95% confidence interval (*p* < 0.05). **d** and **g** Chao1 and Shannon index of the microbiota of different microhabitats from the six rice varieties aggregated from four regions. ns represents no statistical difference based on the *T*-test (*p* < 0.05). **e** and **h** Chao1 and Shannon index of the microbiota of different microhabitats from six rice varieties aggregated from four regions. Means with the same letters are not statistically different based on the *T*-test (*p* < 0.05). **f** and **i** Chao1 and Shannon index of the microbiota of different microhabitats collected from all the rice varieties and regions. Means with the same letters are not statistically different based on the *T*-test (*p* < 0.05). The indices (*F* and *p*-value) are displayed at the bottom of each graph

To comprehensively explore the structure and community composition of the rice-associated microbiota, we first grew three *indica* and three *japonica* varieties in Nanchang (NC) to harvest the parental seeds and then grew these seeds in four geographic locations, Sanya (SY), Langfang (LF), Nanchang (NC), and Xishuangbanna (XB), to examine their microbiomes (Supplementary Fig. 1a). Total DNA from the bulk soil, rhizosphere, root, stem, and seed samples was extracted, amplified using primers for the V3-V4 regions of the bacterial *16S rRNA* gene and sequenced as described in the “Materials and methods” section (Fig. 1a). We generated 249,443,008 raw reads with an average merged read length of 379 bp from 481 samples (20 bulk soil, 107 rhizosphere, 117 root, 106 stem, and 131 seed samples) (Supplementary Table 1). After quality trimming and removal of chimeric and plant reads, we obtained 24,504,476 high-quality reads that were assigned to 26,646 bacterial ASVs (Supplementary Table 2). Moreover, 16S rRNA ASVs were assigned to 28 bacterial phyla and 929 genera (Supplementary Table 2). Rarefaction analysis indicated that our sequencing was sufficient to reveal the true diversity of the samples (Supplementary Fig. 2).

### Diversity and driving factors of rice microbiomes

To evaluate the diversity of the rice-associated microbiomes, *α*-diversity (abundance and richness, Chao1 index; diversity and evenness, Shannon index) was calculated at the ASV level across samples grouped into different varieties, microhabitats, and locations. First, we investigated the microbiomes and physicochemical properties of bulk soil samples from four distinct locations to elucidate the basic effects of geographic environments on the microbial community. Remarkably, the *α*-diversity metrics (Chao1 and Shannon indices) revealed a significant difference in microbial composition among locations, wherein LF and NC had higher abundance and diversity than SY and XB (Chao1, *p* < 0.001; Shannon, *p* < 0.001) (Fig. 1 b and c; Supplementary Table 3). We tested 12 physicochemical properties for all of the bulk soil samples and observed distinct profiles, indicating that the four locations had significant differences in soil fertility (Supplementary Fig. 1b). Redundancy analysis (RDA) indicated that environmental factors significantly shaped the soil microbial communities (permutation test: *p* = 0.005). Two dimensions (RDA1 and RDA2) explained 70.26% of the changes in the soil bacterial community (Supplementary Fig. 1c). The main contributors to these differences were soil ammonium nitrogen (A-N; *p* < 0.0001) and total potassium (TK; *p* < 0.0001). LF and NC had significantly higher soil nitrate nitrogen (NO-N) concentrations, whereas XB had higher concentrations of soil organic matter (OM), organic carbon (OC), and total carbon (TC) (Supplementary Fig. 1c).

Next, we sought to determine the factors that drive the rice microbiome assemblage. We found that the rhizosphere, root, stem, and seed endophytic microbiomes of a single rice variety did not show any significant differences in the *α*-diversity indices at the same location (Supplementary Fig. 3). Furthermore, there were no differences in the *α*-diversity indices across the six rice varieties in the rhizosphere, root, stem, or seed endophytic microbiomes from the four locations (Fig. 1 d and g; Supplementary Table 3), indicating that the microbial diversity was typically independent of the rice variety. However, for both individual rice varieties and aggregates of the six varieties, the *α*-diversity indices of the rhizosphere microbiomes exhibited considerable heterogeneity among the four locations (Fig. 1 e and h, Supplementary Fig. 3; Supplementary Table 3). The trend of the overall *α*-diversity indices of the rhizosphere microbiomes from the four locations was similar to that in the bulk soil, implying that the rhizosphere microbial diversity was influenced by geographic location. In contrast to the bulk soil and rhizosphere, the endophytic microbial diversity of stems, roots, and seeds among the four locations had varied profiles (Fig. 1 e and h; Supplementary Table 3), suggesting that environmental or external influences had little impact on the internal microbial diversity of rice. Notably, the microbial *α*-diversity indices of various plant compartments decreased significantly in the rhizosphere, roots, stems, and seeds, regardless of grouping by rice variety, geographic location, or as the entire collection (*p* < 0.001) (Fig. 1 f and I; Supplementary Table 3). These results suggest that the microhabitat, rather than the geographic location or rice variety, is the primary determinant of microbial diversity.

**Fig. 2:**
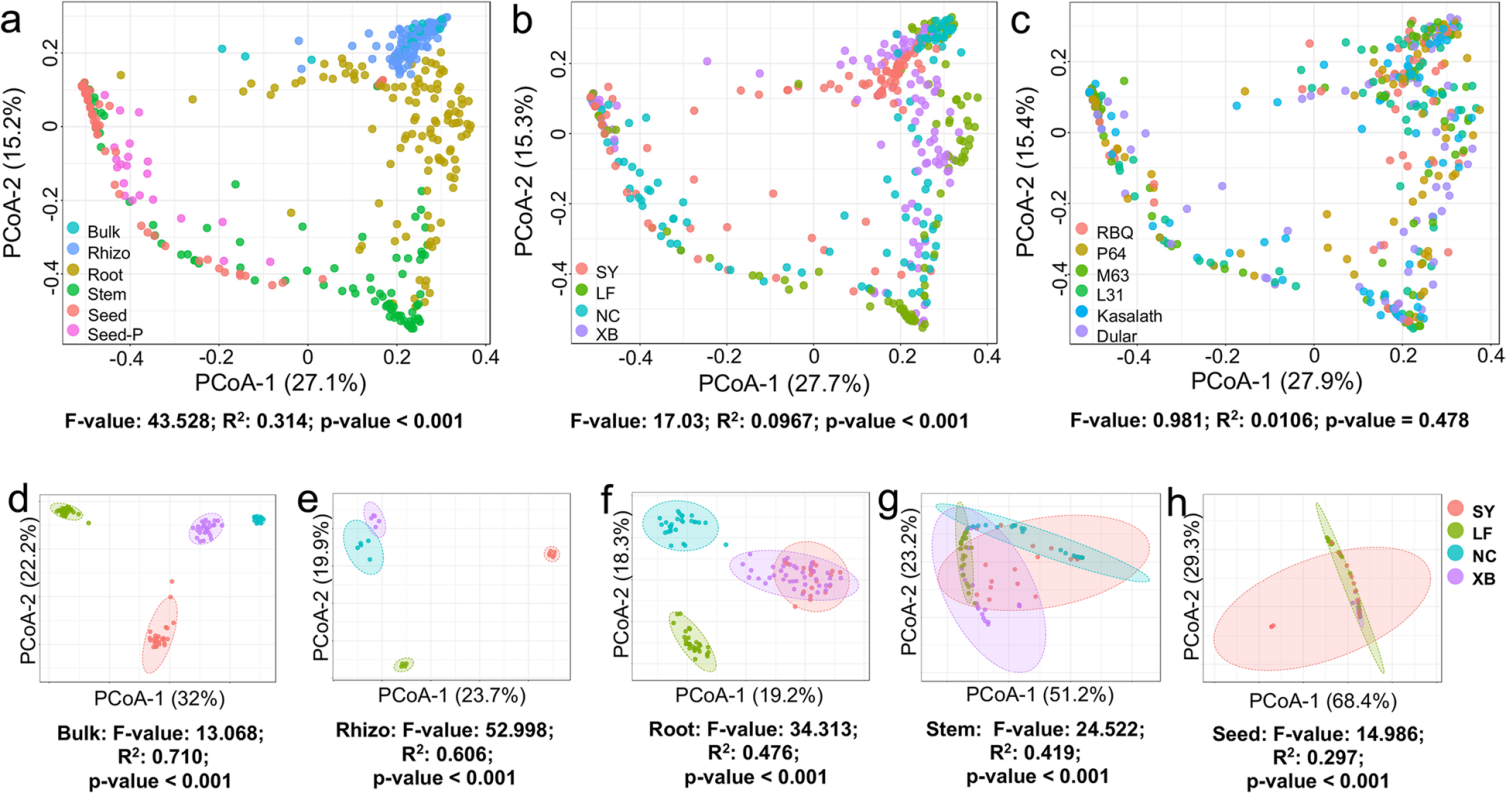
Beta diversity patterns of rice-associated microbiomes. **a** Principal coordinates analysis (PCoA) with Bray-Curtis distances of microbial communities unconstrained by microhabitat, region, and rice variety. PCo-1 and PCo-2 show the first and second components of the PCoA analysis, respectively. **b** PCoA of microbial communities in each microhabitat among the four regions. Regions are highlighted by ellipse and point shape. Significance of the microbial community dissimilarities among different groups is based on PERMANOVA tests. Indices (*F*, *R*^2^, and *p*-value) are displayed at the bottom of each graph

To support our claim, we performed principal coordinate analysis (PCoA) to investigate the internal correlations between these variables (Fig. 2). PCoA demonstrated a significant separation and distribution of the rice microbiomes along the rhizosphere, roots, stems, and seeds (*R*^2^ = 0.314, *p* < 0.001) (Fig. 2a), rather than the geographic locations (*R*^2^ = 0.0967, *p* < 0.001) (Fig. 2b) and rice varieties (*R*^2^ = 0.0106, *p* = 0.478) (Fig. 2c). Remarkably, the seed endophytic pattern of the progeny overlapped significantly with that of the parents (Fig. 2a). Furthermore, PCoA based on Bray–Curtis dissimilarity revealed that the bulk soil microbiomes formed four distinct clusters when colored based on the geographic location (*R*^2^ = 0.710, *p* < 0.001) (Fig. 2d). Despite this, the microbiomes from diverse microhabitats characterized by location began to converge along the rhizosphere, roots, stems, and seeds and overlapped substantially in stems and seeds (Fig. 2 e–h). Taken together, our findings demonstrated that microhabitat was the primary driver of rice microbiome assemblage. Diversity of the rice-associated microbiome declined steadily from far to near the roots, from rice exterior to interior, and from belowground to aboveground niches.

### Taxonomic composition and dynamics of rice microbiota

**Fig. 3:**
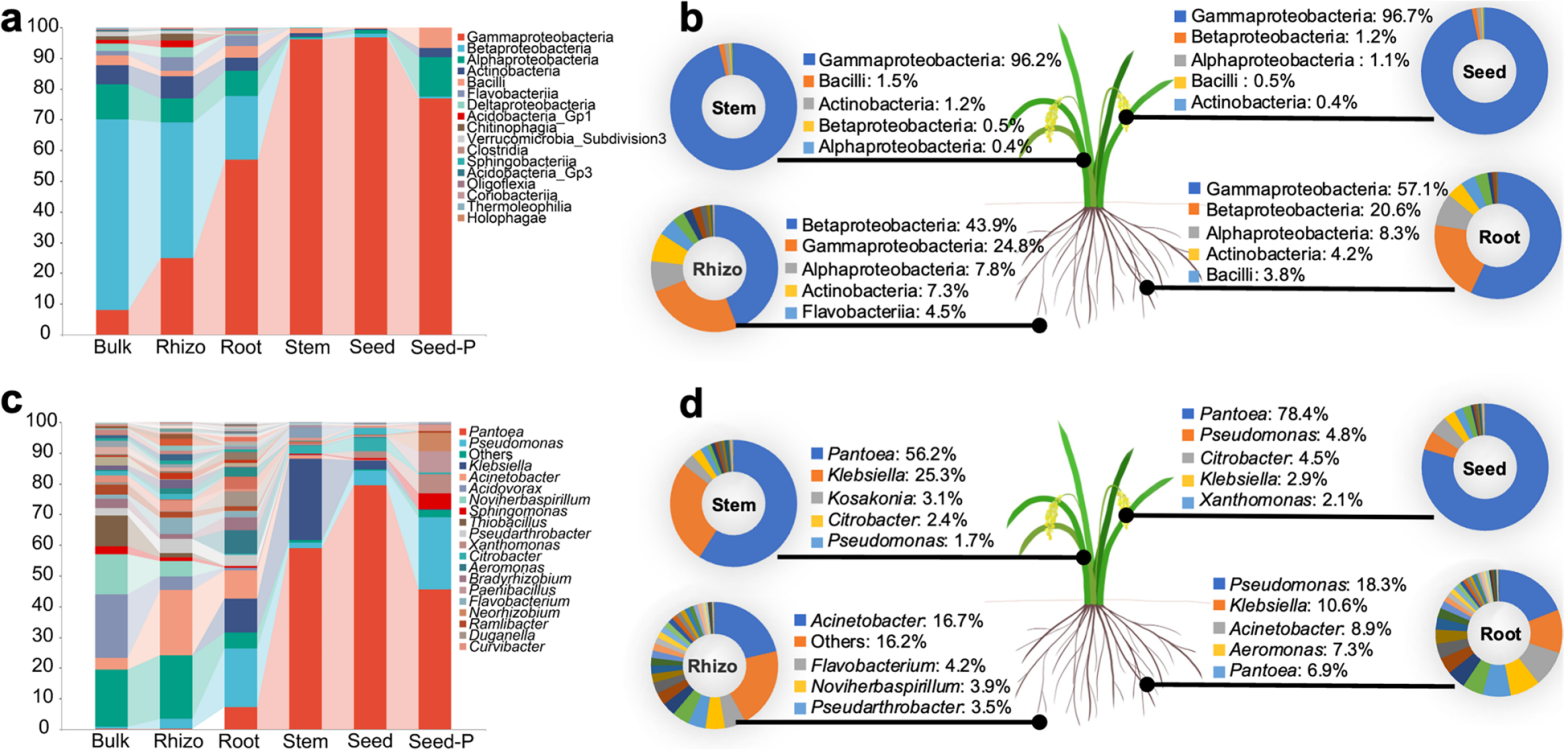
Taxonomic composition of the rice microbiome in each microhabitat. **a** Histograms of class abundances present in bulk soil (bulk), rhizosphere (Rhizo), root endosphere (root), stem endosphere (stem), offspring seed endosphere (seed), and parental seed endosphere (seed-P). **b** Schematic diagram of the top 5 classes of microbiota in each rice compartment. **c** Relative abundances of the most prevalent genera in different microhabitats. **d** Schematic diagram of the top 5 genera of microbiota in each rice compartments

We examined the enrichment and taxonomic differences among rice microbiomes under diverse conditions. The data in Fig. 3, Supplementary Fig. 4, and Supplementary Table 4 reveal that all taxa at various levels were enriched in or depleted in the rice microbiomes. Although *β*-Proteobacteria was the most prevalent bacterial class, accounting for > 20% of the ASVs in each bulk soil samples, the four geographic locations had distinct microbial patterns (Supplementary Fig. 4; Supplementary Table 4). In the SY bulk soil, *β*-Proteobacteria accounted for 57.9% of the ASVs, followed by other classes with < 10% percentage (Supplementary Fig. 4; Supplementary Table 4). In contrast, Actinobacteria was widely distributed in LF and XB, accounting for the second (19.4% of the ASVs) and third (13.3% of the ASVs) most abundant classes in these two locations, respectively, whereas Verrucomicrobia subdivision 3 was abundant in the NC bulk soil, accounting for 12.2% of the ASVs (Supplementary Fig. 4 and Supplementary Table 4). The bacterial class taxonomic distributions in the rhizosphere were similar to those in the bulk soil at each location. For example, *β*-Proteobacteria remained the most common class (Supplementary Fig. 4; Supplementary Table 4). Some classes, on the other hand, were enriched or depleted from the bulk soil to the rhizosphere (Supplementary Fig. 4; Supplementary Table 4). In particular, the proportion of *γ*-Proteobacteria in the rhizosphere increased relative to that in the bulk soil at each location, but the proportion of Actinobacteria in the rhizosphere showed the opposite trend (Supplementary Fig. 4 and Supplementary Table 4).

These alterations were also observed throughout the collection (Fig. 3a; Supplementary Table 4). Remarkably, *γ*-Proteobacteria was the only class that was enriched progressively, whereas a few classes, including Actinobacteria, *α*-Proteobacteria, and *β*-Proteobacteria, were depleted in the bulk soil, rhizosphere, stems, and seeds (Fig. 3 a and b; Supplementary Table 4), indicating that aboveground and rice interior niches are more favorable for *γ*-Proteobacteria survival than the survival of other bacterial classes. Consequently, *Pantoea* and *Xanthomonas*, two representative genera of *γ*-Proteobacteria, became more dominant, whereas *Sphingomonas* from *α*-Proteobacteria and *Acidovorax* from *β*-Proteobacteria became less dominant from the bulk soil to the seed (Fig. 3 c and d; Supplementary Table 4). Moreover, the top five classes or genera in the diverse microhabitats of microbiomes presented dynamic changes, and *γ*-Proteobacteria and *Pantoea* emerged as the most dominant class and genus, respectively, in the offspring seeds (Fig. 3). These findings are consistent with the above observation that microbial abundance and diversity tend to decrease from the bulk soil to the seeds (Fig. 1).

### Bacterial co-occurrence networks and keystone taxa

**Fig. 4:**
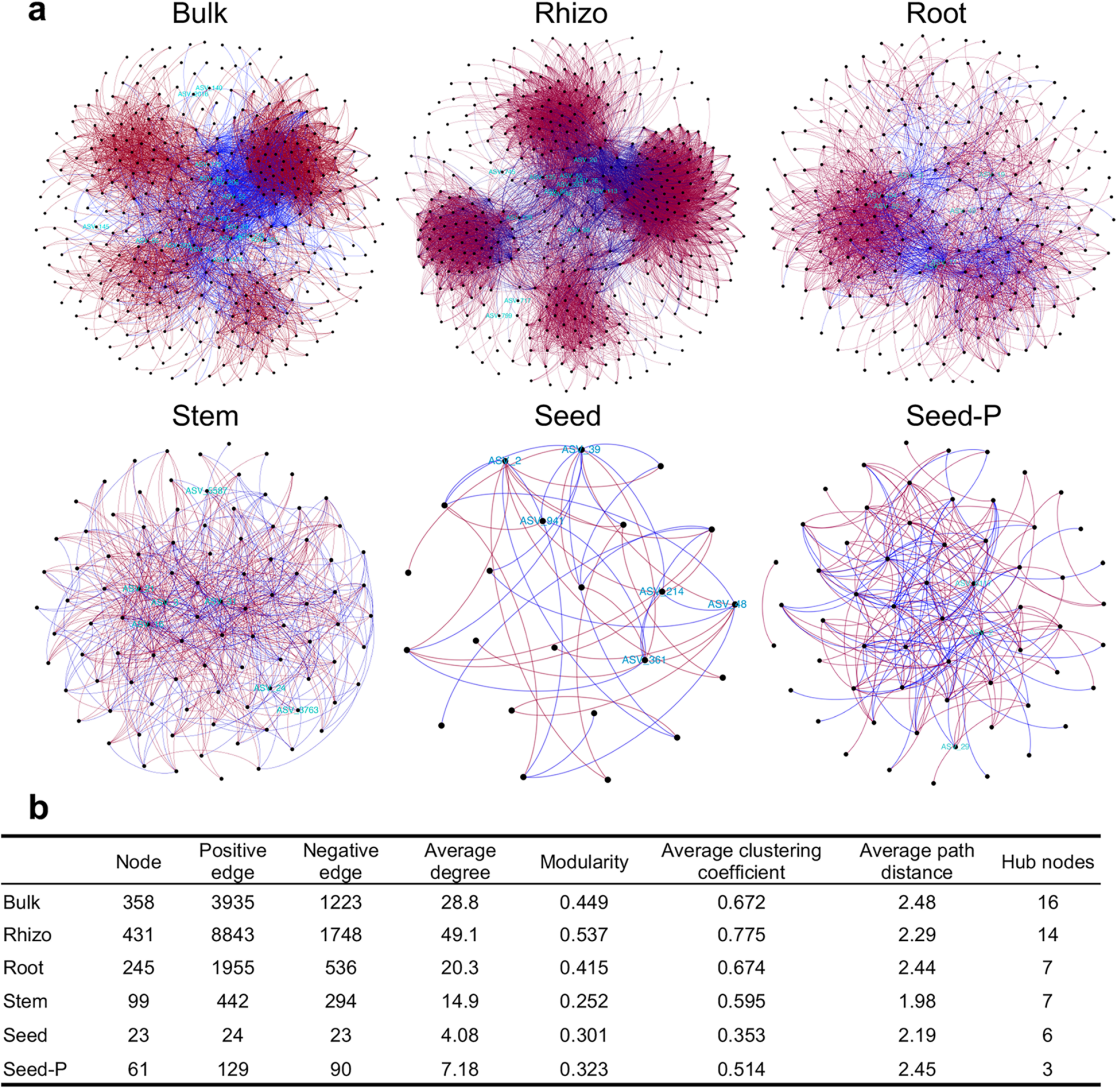
Temporal dynamics of microbial networks. **a** Co-occurrence and mutual exclusion relationships among rice-associated microbiomes across the soil-plant continuum. SparCC algorithm was used to calculate the network at ASV level (abundance > 0.01%) with *p* < 0.05. Each node represents a single ASV, and hub nodes are indicated in cyan. Color of edges indicates the type of the interaction. Red, positive or co-occurrence; blue, negative or mutual exclusion. **b** Major topological properties of co-occurrence networks of each microhabitat. Detailed data is presented in Supplementary Table 5

To further characterize the effects of the complex interactions between bacterial taxa and the microhabitat on rice microbiomes, particularly in endophytes, we assessed the co-occurrence patterns of bacterial communities along the soil-seed continuum. Overall, the network complexity substantially decreased from the rhizosphere to the roots, stems, and seeds (Fig. 4). However, the modularity indices of the bulk soil, rhizosphere, and root were larger (> 0.4) than those of the stem, seed, and parental seed (< 0.4), suggesting that the belowground and root-associated microbiomes had more typical module structures than the aboveground and rice endophytes (Fig. 4). Remarkably, the number of nodes, number of edges, average degree, modularity, and average clustering coefficient were the highest in the rhizosphere, suggesting a highly structured, strongly correlated, and complex network with the most links (Fig. 4). In contrast, offspring seeds had the lowest numbers of nodes and edges, average degree, and average clustering coefficient, indicating a weakly correlated and dispersed network with a few links (Fig. 4). The parental seeds (seed-P) were collected only from NC, which may have resulted in higher topological features than those of the offspring seeds. In the networks, the ratio of positive to negative connections (edges) gradually decreased from the rhizosphere (5.1) to the seed (1.0), indicating more competitive connections in the microhabitat of seeds. Thus, the microhabitats had marked effects on the microbial network.

Microbial communities usually harbor keystone taxa that can be computationally identified as hubs (module hubs and connectors) based on the within-module degree (*Zi*) and among-module connectivity (*Pi*) of ASVs in the networks [23]. Overall, 40 connectors and 13 module hubs were detected in all networks (Supplementary Fig. 5; Supplementary Table 5). Bulk soil and rhizosphere networks had higher numbers of hub nodes, with 17 and 16 hub nodes, respectively, whereas the number of hub nodes successively decreased to six and three in the networks of offspring seeds and parental seeds (Fig. 4b; Supplementary Table 5). Proteobacteria was the dominant keystone taxon in each network, representing 81.25%, 78.57%, and 71.43% of the total hubs in the bulk soil, rhizosphere, and roots, respectively (Supplementary Table 6). Furthermore, all keystone taxa of stems and seeds were classified as Proteobacteria (Supplementary Table 6), indicating the importance of Proteobacteria in the rice interior compartments. Although the seeds had a relatively low number of hub nodes, their highest betweenness centrality indicated the importance of the nodes for network stability and structure (Supplementary Table 5).

### Core rice endophytic microbiota

**Fig. 5:**
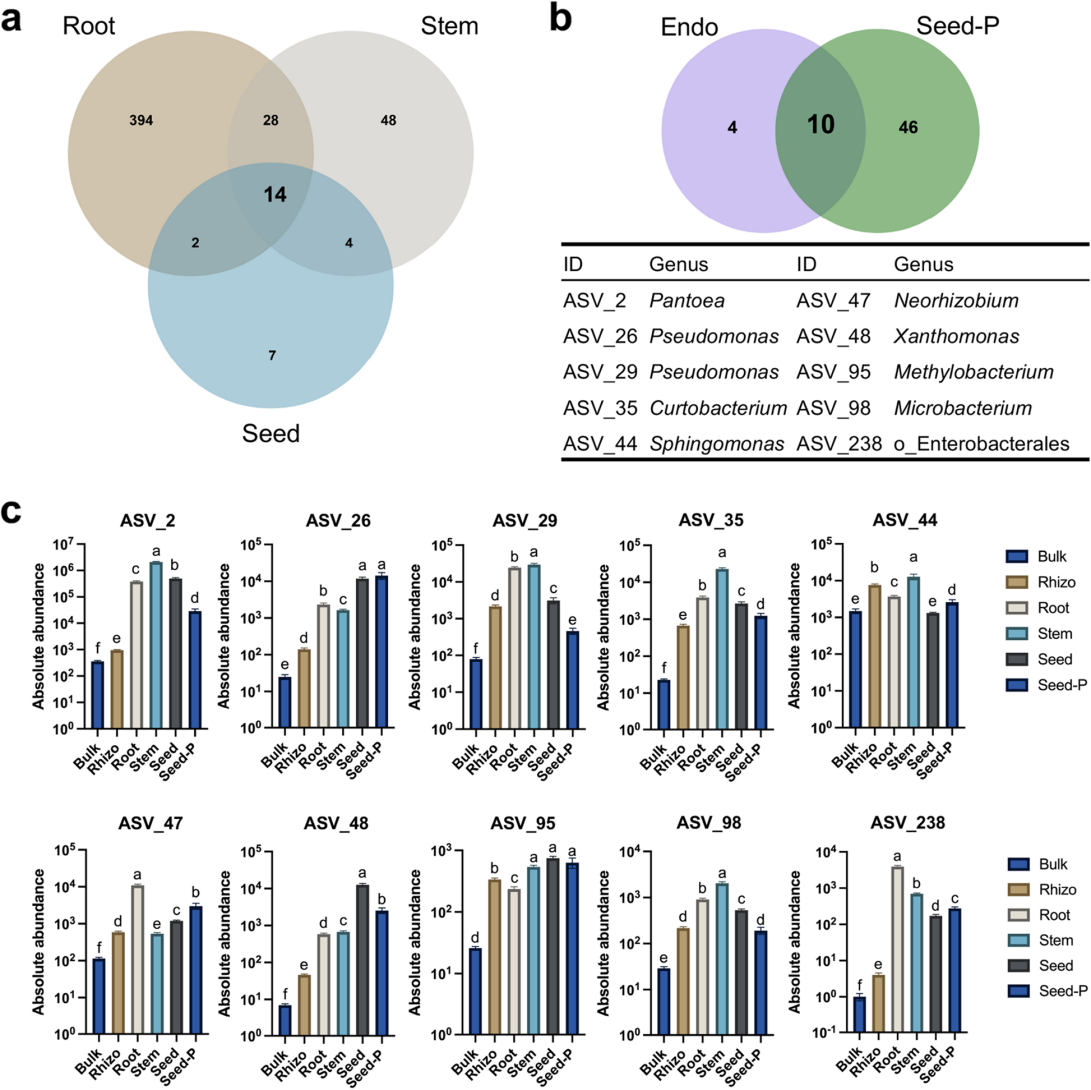
Core rice endophytic microbiota and vertically transmitted taxa. **a** Venn diagram showing that 14 ASVs that coexist in the rice endosphere compartments (root, stem, and offspring seed) at a frequency threshold of 70%. **b** Venn diagram showing that 10 ASVs that are vertically transmitted from the parental seeds (seed-P) to the offspring rice endosphere (endo) at a frequency threshold of 100%. Taxonomy of the ASVs is listed in Supplementary Table 7. **c** Absolute abundance of 10 vertical transmission ASVs in each microhabitat. Sequencing number in ASV table was summed up on microhabitat level with every single replicates. *Y*-axis was log transformed, and statistical significance was determined using one-way ANOVA, Brown-Forsythe, and Welch tests. Letters represent the statistical differences at the 95% confidence interval (*p* < 0.05). Detailed data is presented in Supplementary Table 7.

Although the rice microbiome exhibited dynamic changes depending on the conditions, core microbiome members were ubiquitously present in rice, regardless of the impact of external factors. To elucidate the core microbiome of the rice endosphere, we examined data collected from various locations, rice varieties, and microhabitats. We found that 438, 94, and 27 ASVs were common (70% occurrence) in the collected samples of root, stem, and offspring seed samples, respectively (Fig. 5a). Among these, 10% of the ASVs from roots overlapped with those from stems, and the shared ASV number (42) was 44.7% in stems. Subsequently, 20% of the ASVs from stems overlapped with those from offspring seeds, and the shared ASV number (18) was 66.7% in the offspring seeds. In total, 14 ASVs were identified in all three endophytic rice compartments and were therefore defined as members of the rice core endophytic microbiota, accounting for 51.9% of the ASVs in offspring seeds and were distributed across two phyla, spanning six orders and 13 genera (Fig. 5a; Supplementary Table 7). The most abundant phylum and order of the core endophytes were Proteobacteria and Enterobacterales, accounting for 12 and five ASVs, respectively (Supplementary Table 7). Therefore, we presumed that the shape of the core endophytic microbiome is not completely random. It is partly affected by bacterial properties, host environmental, and metabolic features.

### Evidence for the vertical transmission of seed endophytes

To identify the potential vertically transmitted microbiota, we identified the overlapping ASVs between the core endophytic microbiota and the parental seed endophytes. We observed 10 ASVs overlapping between the two groups, and they were assigned to nine genera, which suggests the possible occurrence of inheritance across generations of rice core endophytes (Fig. 5b). Among these, shared ASVs accounted for 71.4% of the core rice endophytic taxa. Remarkably, the absolute abundances and occurrence frequency of ASV_2 (*Pantoea*), ASV_26 (*Pseudomonas*), ASV_48 (*Xanthomonas*), and ASV_238 (*o_Enterobacterales*, unclassified genus) in the root, stem, and parental and offspring seeds were significantly higher than those in the bulk soil and rhizosphere (Fig. 5c and Supplementary Fig. 6). This result suggests that these four ASVs are most likely to be the vertically transmitted taxa. We demonstrated that only a few specific microorganisms were transmitted, indicating that a filtering process occurred during the transmission [14, 24].

**Fig. 6:**
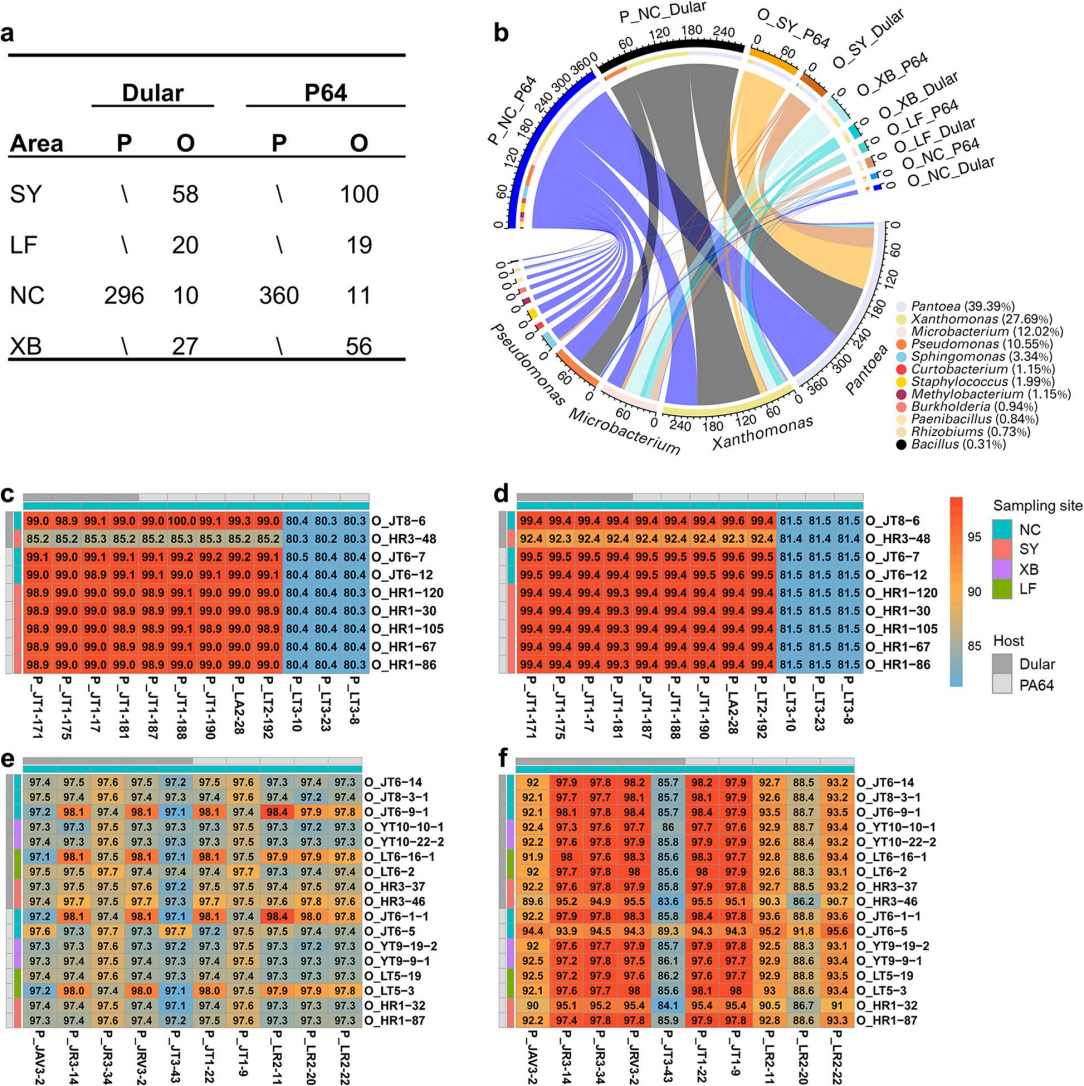
Identification of culturable vertically transmitted rice seed endophytes. **a** Number of culturable bacteria from parental (P) and offspring (O) seeds of two rice varieties (Dular and P64) harvested from four regions (SY, LF, NC, and XB). **b** Circular representation of the proportional structure of bacterial strains at genus level associated with the isolation groups listed in **a**. Visualization was rendered using the open-source software Circos. **c** Heatmap of reciprocal average nucleotide identity (ANI) of the *Pantoea* isolated from parental (P_) and offspring seeds (O_). ANIs were calculated using FastANI. Colored boxes indicate similarities scaled from low (blue) to high (red). **d** Heatmap of reciprocal concatenated core genome identity of the *Pantoea* isolated from parental (P_) and offspring seeds (O_). Analysis was based on reciprocal global alignment of concatenated 2505 single-copy gene sets of the 21 *Pantoea* strains. Colored boxes indicate similarities scaled from low (blue) to high (red). **e** Heatmap of reciprocal ANIs of the *Xanthomonas* isolated from parental (P_) and offspring seeds (O_). Annotation is the same as in **e**. **f** Heatmap of reciprocal concatenated core genome identity of the *Xanthomonas* isolated from parental (P_) and offspring seeds (O_). The analysis was based on reciprocal global alignment of concatenated 2508 single-copy genes sets of the 27 strains of *Xanthomonas* strains. Colored boxes indicate similarities scaled from low (blue) to high (red)

**Fig. 7:**
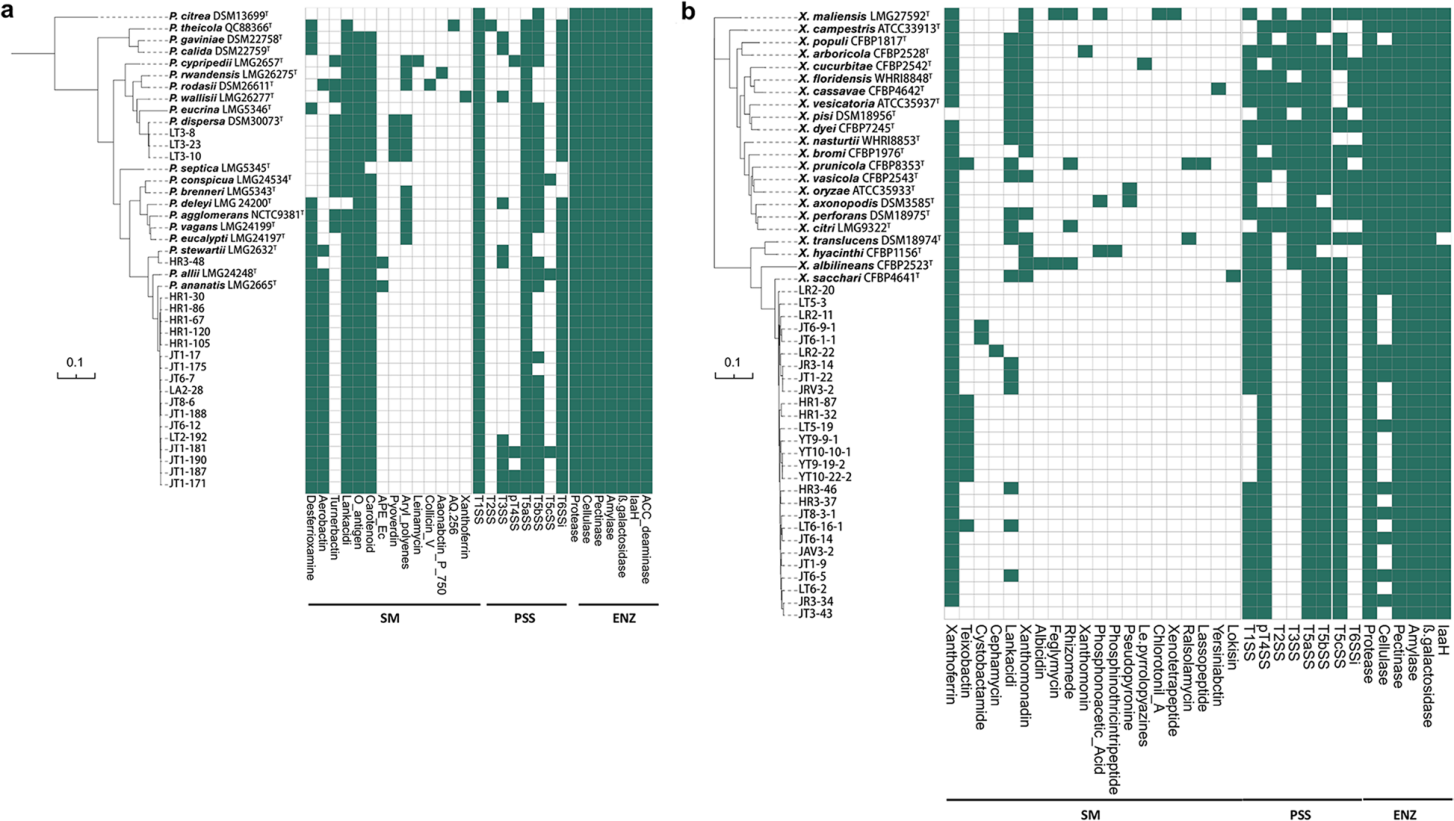
Phylogenetic analysis and characterization of vertically transmitted rice seed endophytes. **a** Maximum likelihood phylogeny of 21 isolates of *Pantoea* with 20 type strains based on the concatenated multiple alignment of the 1258 single-copy orthologous genes. Shimodaira-Hasegawa test support values (percentage of 1000 replications) greater than 75% were indicated at branching nodes. Selected biosynthetic, secretion system-related and catabolic genes or gene clusters were predicted using the genome sequences of the *Pantoea* isolates. **b** Maximum likelihood phylogeny of 27 isolates of *Xanthomonas* with 22 type strains based on the concatenated multiple alignment of the 892 single-copy orthologous genes. Selected biosynthetic, secretion system-related, and catabolic genes or gene clusters were predicted using the genome sequences of the *Xanthomonas* isolates. SM, secondary metabolites; PSS, protein secretion systems; ENZ, digestive enzymes. Dark green and brown boxes represent the presence and absence of a gene or gene cluster within a genome, respectively. The bootstrap values greater than 80 are not displayed

To obtain the vertically transmitted taxa, we performed high-throughput isolation and identification of endophytic bacteria from seeds. Collectively, we obtained 957 bacterial isolates from the parental and offspring seeds of two rice varieties (PA64 and Dular) harvested from four different geographic locations (Fig. 6a). The *16S rRNA gene* sequencing and analysis showed that the isolates were assigned to three phyla, five classes, and 12 genera (Fig. 6b). *Pantoea* and *Xanthomonas* were the two most abundant genera, representing 39.39% and 27.69% of all the isolates, respectively (Fig. 6b). The *16S rRNA gene* sequences of 21 *Pantoea* and 27 *Xanthomonas* strains were identical to those of ASV_2 and ASV_48, respectively (Fig. 6b). Among them, nine *Pantoea* and 17 *Xanthomonas* strains were isolated from the offspring seeds, and the rest were from the parental seeds (Fig. 6 c and e). We performed draft genome sequencing of these bacteria to explore their shared identities. The average genome sizes of the *Pantoea* and *Xanthomonas* strains were 5.07 and 4.84 Mb, respectively (Supplementary Table 8). ANI and core genome phylogenetic analysis of *Pantoea* isolates with type strains indicated that the isolates belonged to three species: *P. ananatis*, *P. dispersa*, and *P. stewartia* (Fig. 7a; Supplementary Fig. 7a). Similarly, all *Xanthomonas* isolates were classified into one species, *X. sacchari* (Fig. 7b; Supplementary Fig. 7b). Overall, all eight *Pantoea* strains from the offspring seeds showed ≥ 98.9% ANI and ≥ 99.3% concatenated core genome identity with the nine strains from the parental seeds (Fig. 6 c and d). Notably, *P. ananatis* JT8-6 from the offspring seeds of Dular exhibited a 100% ANI with *P. ananatis* JT1-188 from the parental seeds of PA14. Among the *Xanthomonas* isolates, four strains (JT6-9-1, LT6-16-1, JT6-1-1, and LT5-3) from the offspring seeds had ≥ 97.9% ANI with four strains (JR3-14, JRV3-2, JT1-22, and LR2-11) from the parental seeds (Fig. 6e). Furthermore, the four offspring strains showed ≥ 98.0% concatenated core genome identity with JRV3-2 and JT1-22 (Fig. 6f). The extremely high identity between the isolates from parental and offspring seeds suggests the vertical transmission of seed endophytes at the strain level.

### Genome mining and phenotypic characterization of vertically transmitted seed endophytes

To elucidate the potential functions of the vertically transmitted taxa, we performed genome-mining analysis of all of the sequenced endophytic *Pantoea* and *Xanthomonas* strains. We calculated the number of pan and core genes in *Pantoea* and *Xanthomonas* strains, respectively. There were 3091 and 2627 core genes in isolated *Pantoea* and *Xanthomonas* strains, respectively, with the pan genes accounting for 26.8–39.8% of the whole genome (Supplementary Fig. 8 a and c). The pan-genome accumulation curves with the heaps law model index indicated an open-pan genome among *Pantoea* and *Xanthomonas* strains (Supplementary Fig. 8 b and d). The large number of core genes derived from pan-genome analysis prompted us to look at their functional classification to obtain clues for vertical transmission characteristics. The core genomes of *Pantoea* and *Xanthomonas* strains were then annotated using the Clusters of Orthologous Genes (COG) and Kyoto Encyclopedia of Gene and Genomes (KEGG) databases, and we found that the E (amino acid transport and metabolism) and G (carbohydrate transport and metabolism) terms were distinctively enriched in COG annotation in both *Pantoea* and *Xanthomonas* core genomes and highly correlated with the top 2 KEGG annotated terms “Carbohydrate_metabolism” and “Amino_acid_metabolism” (Supplementary Fig. 9). Subsequently, we divided these characteristics into three categories: secondary metabolites, protein secretion systems, and enzymes (Fig. 7). In silico prediction revealed that all *Pantoea* strains contained plant growth promotion-associated genes encoding 1-aminocyclopropane-1-carboxylic acid (ACC) deaminase and indoleacetamide hydrolase (*iaaH*) (Fig. 7a). Moreover, all *Pantoea* strains possessed genes encoding various digestive enzymes, such as dextranase, *β*-galactosidase, pectinase, cellulase, and amylase (Fig. 7a). All the *Pantoea* strains had a type 1 secretion system (T1SS), T5aSS and T6SS, whereas only a few strains had T2SS, T3SS, T4SS, T5bSS, and T5cSS (Fig. 7a). Although the three species of *Pantoea* had distinct patterns of secondary metabolites, the overall metabolite profile generated by antiSMASH was fairly rare and was composed primarily of iron chelation-associated desferrioxamine, aerobactin, turnerbactin, and pyoverdine rather than the antimicrobial biosynthetic gene clusters (BGCs). *Xanthomonas* strains also had the *iaaH* gene and abundant digestive enzyme-encoding genes for *β*-galactosidase, amylase, and pectinase (Fig. 7b). However, the isolated *Xanthomonas* strains only contained only T1SS, T4SS, and T5SS rather than the pathogenic (type strain) T3SS and T6SS (Fig. 7b). The isolated strains had a very limited secondary metabolite profile, which consisted merely of a siderophore-like xanthoferrin and an inconclusive teixobactin, while the type strains mostly contained lankacidi and xanthomonadin (Fig. 7b). The small genomes and streamlined metabolism of *Pantoea* and *Xanthomonas* may assist in the adaptation to life in the rice seed interior compartment.

To determine whether the vertically transmissible isolates truly possessed plant growth-promoting properties, we determined a few representative characteristics of the selected strains of *Pantoea* and *Xanthomonas* (Supplementary Table 9). We found that all the strains had cellulase activity and produced indole-3-acetic acid (IAA), which corresponded to the results of genome mining. Additionally, we tested the phosphorus solubilization activity and found that most of the strains, including *Pantoea* and *Xanthomonas*, could solubilize inorganic and organic phosphates. We also assessed whether *Pantoea* and *Xanthomonas* could antagonize the major pathogens of rice diseases, such as *Magnaporthe oryzae* and *X. oryzae* pv. *oryzae*. However, we were unable to observe the growth inhibition of the two rice pathogens by most of the bacterial strains, except for five *Pantoea* strains that showed moderate antagonistic activity, which can be explained by the rare antimicrobial metabolites predicted by genome mining.

## Discussion

Stable plant-microbe interactions are contingent on continuous coexistence over generations [25, 26]. Seed endophytes are of particular interest because they can be transmitted from parent to progeny [1, 7]. To identify the core vertically transmitted taxa of the rice microbiome, we collected and determined the microbiome from five microhabitats of six rice varieties with two generations from four geographic locations (Supplementary Fig. 1a). Our results demonstrated that the rice microbiome was largely shaped by the microhabitats rather than the site-dependent environmental factors and host varieties. We further showed that rice compartments had a strong and direct effects on endophytic bacterial diversity and network complexity, with the selection pressure sequentially enhanced, and the taxa gradually filtered and enriched from the rice exterior to interior and from belowground to aboveground niches. This could be explained by other findings or hypotheses regarding intensive selection pressures caused by the host immune system and physical and biochemical barriers in the plant interior compartments [27–29]. By analyzing over two successive generations, we identified the vertically transmission profile and keystone taxa of the rice microbiome using multiple bioinformatic methods, facilitating the isolation of culturable seed endophytes and exploration of their potential functions in plant-microbiome interactions. These findings provide comprehensive and empirical evidence for the predicted vertical transmission of the seed endophytic microbiome and coevolution of the host and microbes.

A point worth highlighting is the dynamic change in the *β*- and *γ*-Proteobacteria abundance between the rice exterior and interior microhabitats (Fig. 3). *β*-Proteobacteria, which were widely distributed and abundant in the bulk soil and rhizosphere in our study, represented specific taxa that were better adapted to natural environments, where they were involved in nutrient cycling, including the regulation cycles of carbon (C), nitrogen (N), iron (Fe), and sulfur levels (S) [30–34]. In roots, stems, and seeds, *γ*-Proteobacteria became the most dominant taxa, and Enterobacteriaceae and Pseudomonadaceae within *γ*-Proteobacteria were significantly enriched in plant interior compartments (Supplementary Table 7). Further metagenomic studies can shed more light on the seed microbiome and provide a better understanding of the endophytic microbiota present in seeds with regard to both the taxa and their phenotypic characteristics. At the genus level of classified taxa, *Klebsiella*, *Pseudomonas*, and *Citrobacter* were constant inhabitants e in the rice endosphere with high abundance (Fig. 3) and were capable of fixing atmospheric nitrogen, producing phytohormones that benefit plant growth, and modulating host fitness, pathogen suppression, and plant tolerance to stress [35–41]. Among the genera of *γ*-Proteobacteria, the proportions of *Pantoea* and *Xanthomonas* tended to increase the fastest from roots to seeds, and *Pantoea* had a strong predominance (78.4%) in seeds. Ferreira et al. reported that a gfp-labeled *Pantoea* strain could colonize the embryo and move into the intercellular spaces of the root and interior of the stem xylem vessels [42]. Besides, Johnston-Monje and Raizada (2011) showed that a *gfp*-labeled seed-borne *Pantoea* strain injected into the stem of maize plants was able to move systemically through the vascular tissues and was present in the metaxylem vessels [43]. Together, our findings on vertically transmitted taxa from the core rice endophytes supported the hypothesis that these taxa remained inside the seed to be spread evenly through plant growth or move within the plant tissue [44].

Culture collection of rice seed endophytes represented most of the vertically transmitted taxa, including the dominant genera *Pantoea* and *Xanthomonas*. *Pantoea* is considered an endophytic genus in various plant hosts as described above and in other studies [45–48]. Although some species of *Pantoea* can cause plant diseases [49], many studies have demonstrated that *Pantoea* species are mostly mutualists or commensals, rather than pathogens [25, 48, 50]. However, *Xanthomonas* species are usually recognized as plant pathogens that obligately rely on the T3SS and T6SS to cause diseases in hundreds of plant species [51–53]. Recently, a whole-genome analysis of type strains and completely sequenced genomes of *Xanthomonas* spp. revealed that *Xanthomonas sacchari* was the only species that did not rely on T3SS and T6SS [52]. *X. sacchari*, coincidentally, was the vertically transmitted endophytic *Xanthomonas* species discovered in our study. Intriguingly, we found that both *Pantoea* and *Xanthomonas* isolated from seeds carried some common features via in silico prediction. Seed-borne *Pantoea* and *Xanthomonas* strains had streamlined genomes (< 5 Mb) and simplified metabolism, with scarce antimicrobial compounds and few protein secretion systems (Fig. 6 d and h; Supplementary Table 8). It is reasonable to assume that the seed interior compartment is a restricted and invariant reservoir in which endophytes maintain the greatest fitness and evolutionary success with the lowest metabolic costs [1, 24, 54]. We noticed that all the isolates encoded plant cell wall-degrading enzymes, such as cellulase and pectinase, which were important for colonizing seeds, spreading systemically, and ultimately reaching the fruits and offspring seeds [55, 56]. The strains of the two genera also possessed amylase homologs, which use starch and resume growth after long-term survival inside seeds [57]. In addition to the characteristics that benefit their own survival, the isolates produce plant growth-promoting substances such as auxins and ACC deaminase, which enable the plant to pass bacteria with beneficial characteristics to their offspring [11, 58]. Phosphorus is one of the most limited but critical plant growth nutrients, primarily present in insoluble forms, and phosphate-solubilizing rhizobacteria play a critical role in converting phosphorus to available forms [59]. Most of the *Pantoea* and *Xanthomonas* isolates exhibited significant phosphate-solubilizing activity, which suggests the potency of the nutrient conversion capacity of the vertically transmitted seed endophytes. Although a few antimicrobial BGCs were discovered in the isolates, a chemically diverse set of siderophores was also present in the genomes, which compete with the pathogens for iron [41, 60, 61]. All of these findings highlight the significance of the core seed microbiota in ensuring the best-matching microbial symbionts for the next generation. Further genomic and genetic investigation of vertically transmitted rice endophytes should be carried out to unravel the molecular mechanisms contributing to their colonization, association, and coevolution with hosts.

## Conclusion

Our data suggest that the influence of microhabitat on rice-associated microbiota is greater than that of plant variety and soil type in some cases. Identification of the core endophytes, network hubs, and vertically transmitted microbiota provides critical information and lays a theoretical foundation for developing an effective strategy for the future manipulation of seed-associated microbiomes to improve the plant fitness. Remarkably, attractive characteristics such as streamlined genomes and low-complexity metabolic networks of seed endophytes can be exploited in the adaptation of cell factory systems or chassis cells for agricultural, industrial, and environmental applications.

## Materials and methods

### Experimental setup and sampling

In 2016, six rice cultivars (RBQ, L31, M63, P64, Dular, and Kasalath) were bred in Nanchang (NC), Jiangxi, China (115°57′ 34″ E, 28°33′ 9″ N), and seeds were collected as the parental generation. The seeds were harvested and planted in four regions in China: Sanya (SY), Hainan (109°30′ 28″ E, 18°15′ 15″ N), Langfang (LF), Hebei (116°36′ 8″ E, 39°30′ 28″ N), NC and Xishuangbanna (XB), and Yunnan (100°47′ 25″ E, 21°59′ 47″ N). Each area had five replicates, and each replicate contained a section of 19.5 m^2^ (3 m × 6.5 m). Samples were collected from five microhabitats: bulk soil (bulk), rhizosphere (Rhizo), root endosphere (root), stem endosphere (stem), and seed endosphere (seed). The “S”-shaped sampling method was used to collect 10 cm bulk soil [62]. Rice roots were cut with sterile scissors, and the 1–2-mm-thick soil layer surrounding the root was defined as the rhizosphere soil. Rhizosphere soil was collected via centrifugation at 12,000 rpm for 10 min and stored at −80 °C and 4 °C for further processing, respectively.

The soil total nitrogen (TN), ammonium nitrogen (A-N), nitrate nitrogen (NO-N), available nitrogen (K-N), organic matter (OM), total potassium (TK), available potassium (AK), total phosphorus (TP), available phosphorus (AP), total carbon (TC), organic carbon (OC), and pH were measured according to the soil quality guidelines [63].

### Isolation of culturable bacteria

The parental seeds were simultaneously collected from two rice cultivars, Dular and P64, in NC. Harvested seeds were defined as offspring rice seeds. All mature seeds were randomly selected in the field and sealed immediately in sterilized plastic bags at 4 °C until further processing.

Parental and offspring seeds were used to isolate the endophytic bacteria. Five grams of seeds of each cultivar was dehusked first. The surface sterilization method followed that of Verma, with minor modifications [64]. Firstly, the dehusked seeds were rinsed twice with sterile water and treated with 70% ethanol for 3 min. Subsequently, the seeds were sterilized with 1% (available chlorine) sodium hypochlorite for 1 min and 70% ethanol for 30 s and washed thrice with sterile water. The remaining disinfectant on the surface of the seeds was removed with sterile filter paper, and the seeds were rinsed thrice with sterile water. Finally, any remaining water on the surface of the sterile seeds was removed using sterile filter paper. The efficacy of surface sterilization was verified by inoculating 100 μL of sterile water from randomly selected samples into the tryptic soy agar (TSA). The plates were incubated at 30 °C and observed for 5 days to check for microbial growth. Surface-sterilized seeds were ground into powder in a sterile mortar. The powder was then transferred into 50-mL sterile water tubes and maintained at 28 °C and 200 rpm for 30 min. The homogenate was serially diluted in 9 mL sterile deionized water. One-hundred microliters of the homogenate from each dilution (up to 10^−6^) was plated in triplicate on three different culture media: Reasoner’s 2A agar (R2A), A15 nitrogen-free medium (A15), and TSA [65]. Agar plates were incubated at 30 °C for 7 days. All the purified colonies were stored at −80 °C with 20% (v/v) glycerol for long-term storage.

### Illumina and 16S rRNA gene sequencing

Total genomic DNA was extracted using a FastDNA Spin Kit for Soil (MP, USA) according to the manufacturer’s instruction. Then, DNA was examined using 1.0% (v/v) agarose gel electrophoresis and quantified using a NanoDrop One Spectrophotometer (Thermo Scientific, USA). Isolated DNA was stored at −80 °C until further analysis.

For *16S rRNA* gene libraries, the V3-V4 region was amplified using the universal primers 534f (5′-CCAGCAGCCGCGGTAAT-3′) and 783r (5′-ACCMGGGTATCTAATCCKG-3′) [66]. Amplification was performed using the following polymerase chain reaction (PCR) program: 95 °C for 3 min, followed by seven cycles of 95 °C for 45 s, 65 °C for 1 min (decreasing at 2 °C per cycle), and 72 °C for 90 s; secondly, 30 cycles of 95 °C for 15 s, 50 °C for 30 s, and 72 °C for 30 s; and, finally, 72 °C for 5 min as a final extension. PCR amplifications were performed using the Phanta Max Master Mix kit P515 (Vazyme, China). The PCR products were examined using 1.0% (v/v) agarose gel electrophoresis. Amplicon sequencing was performed at Novogene Co., Ltd. on the Illumina HiSeq P250 platform with 250 bp paired-end technology.

The resulting sequences were demultiplexed and quality filtered using Vsearch on the Galaxy platform (version 2.7.2) [67]. Paired-end reads were checked using FastQC (version 0.10.136) [68] and merged using the Vsearch script fastq_mergepairs command. After removing barcodes and primers, low-quality reads were filtered, and nonredundant reads were identified. The zero-radius OTUs (zOTUs/ASV) table was generated using UNOISE3 [69]. After removing the ASVs not present in at least 5% of all samples, we obtained 249,443,008 raw reads with an average length of 379 bp from 481 samples in total (20 bulk soil, 107 rhizosphere, 117 roots, 106 stems, and 131 seed samples). After quality trimming, the taxonomy of each sequence was analyzed by RDP Classifier [70] against the SILVA small subunit rRNA database (version 138) using a confidence threshold of 0.7 [71]. We obtained 24,504,476 high-quality reads that were assigned to 26,646 bacterial ASVs after removing ASVs taxonomically classified as mitochondria, chloroplasts, or *Archaea*. Finally, the 16S rRNA sequences were assigned to 28 bacterial phyla, 929 genera, and 26,646 ASVs.

To identify the 957 bacterial isolates, we amplified the *16S rRNA* genes of the strains using the universal primers 27f (5′-AGAGTTTGATYMTGGCTCAG-3′) and 1492r (5′-TACGGCTACCTTGTTACGACTT-3′) [72] and sequenced the fragments using the primer 1492r in Sanger sequencing approach. Sequences were identified by aligning the EzBioCloud’s identification service using similarity-based searches [73].

### Bioinformatic analysis

Downstream analysis was performed using the MicrobiomeAnalyst online pipeline [74] marker data profiling (MDP) and shotgun data profiling (SDP) modules and R platform (version 4.1.0). All the samples were rarefied to the sample with the least number of sequences (2000 read counts) before downstream analyses.

### Diversity analysis

Alpha-analysis was carried out based on Chao1 and Shannon diversity measures at the ASV level. Beta-analysis was carried out using Bray-Curtis dissimilarity at the ASV level. Permutational multivariate analyses of variance analyses were performed using the “Adonis” function implemented in the vegan package in R [75]. RDA was performed using R script “vegan” (Version 2.5–7) [75].

### Taxonomic composition

Taxa composition was generated at the class and genus levels for all six microhabitats (bulk, rhizosphere, root, stem, seed, and seed-P), and the top five classes or genera accounting for the highest proportions were calculated. A condition of minimum count = 1, prevalence in samples > 10%, and merging all taxa with counts < 10 were used, and six bacterial phyla and 124 genera were filtered for taxonomic composition analysis. For class level taxa, small taxa were merged into “others” with the ASV counts < 10. For genus level taxa, distribution of bacteria with top 50 items was determined, and the rest of genus was merged into “others.” Only the top 20 items were labeled. The distribution of isolates was visualized by Circos plot created using the circlize package in R [76].

### Core taxa selection

We used the EVenn online tool to visualize the ASVs that overlapped among all plant compartments and soils [77]. Overlapping ASVs were defined as those detected in at least one sample in each compartment. The sequencing number in the ASV table was summed at the compartment level with every single replicates. *Y*-axis was log transformed, and statistical significance was determined by two-way analysis of variance (ANOVA). Furthermore, abundance occupancy analyses were conducted at a threshold of 70% to identify the core ASVs across the environmental gradients.

### Network construction

These are features of the global network of rice-associated bacterial communities across the six sampling microhabitats. SparCC algorithm was used to calculate the network at the ASV level with an abundance of > 0.01% and *p* < 0.05. Only ASVs detected in > 25% of the samples were used for network construction. Random matrix theory (RMT) was used to automatically identify the appropriate similarity threshold prior to network construction. All analyses were performed using the molecular ecological network analysis (MENA) pipeline (http://ieg2.ou.edu/MENA/) [23, 78]. The networks were graphed using Gephi 0.9.2 [79]. The modularity of each network was characterized using a greedy modularity optimization method. Furthermore, the within-module connectivity (*Zi*) and among-module connectivity (*Pi*) of each node were calculated to classify the putative keystone species in the network. Node topologies were organized into four categories: module hubs (highly connected nodes within modules, *Zi* > 2.5), network hubs (highly connected nodes within the entire network, *Zi* > 2.5 and *Pi* > 0.62), connectors (nodes that connect modules, *Pi* > 0.62), and peripherals (nodes connected within modules with few outside connections, *Zi* < 2.5 and *Pi* < 0.62). All plots were generated using ggplot2 (version 3.3.2) [80].

### Genome sequencing, assembly, and annotation

Genomic DNA of all strains (*Pantoea* = 21, *Xanthomonas* = 27) was extracted using a HiPure Bacterial DNA Kit (Magen Bio, Guangzhou, China), according to the manufacturer’s protocols, and quantified using a Qubit 3.0 fluorometer (Invitrogen, Carlsbad, CA, USA). Qualified genomic DNA (> 10 nM) was selected for short-read sequencing (2 × 150 bp) using the Illumina MiSeq platform (Illumina, San Diego, CA, USA). Genomes were assembled, predicted the open reading frames were predicted, and annotated with the PGCGAP pipeline (version 1.0.28) [81]. All analyses were performed using the default parameters.

### Functional genome analysis

Secondary metabolism analysis was performed using the Antibiotics and Secondary Metabolite Analysis Shell pipeline (antiSMASH, version 6.0.1) with default parameters [82]. Briefly, the detection strictness was “relaxed,” and all “extra features,” including “ClusterBlast,” “KnownClusterBlast,” “SubClusterBlast,” “Cluster Pfam analysis,” “Pfam-based GO term annotation,” and “ActiveSiteFinder,” were employed for biosynthetic gene cluster (BGC) border prediction and analysis.

Genomes were screened for the presence of secretion systems using MacSyFinder (version 2.5.1) with the “TXSScan” models with command “−db_type ordered_replicon all” [83].

To prediction of cellulase, *β*-galactosidase, indole-3-acetyl-aspartic acid hydrolase (IaaH), and 1-aminocyclopropane-1-carboxylic acid (ACC) deaminase activities; all reference sequences were downloaded from the UniProt database. Blastp (version 2.12.0) was used to search each proteome for homologs with a score of *e*-value ≤ 1e-5 and ≥ 60% alignment length [84]. However, it should be noted that the protein hits in each strain did not necessarily indicate the presence of the corresponding function.

### Phylogenomic reconstruction

We inferred the COGs of the strains (*Pantoea* = 41, *Xanthomonas* = 49) using OrthoFinder (version 2.5.4) [85]. Next, 1258 gene families and 892 gene families exclusively containing single-copy genes present in all genomes separately were selected as potential phylogenetic markers. A concatenated alignment of all markers was built with MAFFT (version 7.475) [86], and phylogenomic trees were constructed with FastTree (version 2.1.10) [87]. The phylogenomic tree was midpoint rooted and visualized using the online tool, Interactive Tree of Life (iTOL) [88].

### Core and pan-genome analyses

Homologous genes were clustered into gene families for all isolated strain genomes (*Pantoea* = 21, *Xanthomonas* = 27) and identified using OrthoFinder software with default parameters respectively. The single-copy core genome sets were extracted from the OrthoFinder output. COG and KEGG pathway annotations were performed using the Blastp software (version 2.2.25) against the COG [89] and KEGG [90] databases. Rarefaction curves for the core and pan-genomes of *Pantoea* and *Xanthomonas* were individually estimated using PanGP [91].

### Heatmap construction of ANI and reciprocal concatenated core genome

Pairwise genomic similarities between parental and offspring isolates (*Pantoea*: 12 vs 9, *Xanthomonas*: 10 vs 17) were calculated using FastANI (version 1.32) [92]. Pairwise core genome identities were calculated using the EMBOSS stretcher tool (version 6.6.0) [93]. Pairwise genomic similarities and core genome identities were then drawn in R (version 4.1.0) package “pheatmap” (https://cran.rstudio.com/web/packages/pheatmap/index.html).

### Plate assays for functional bacteria

Isolated strains were tested for plant growth-promoting traits. Assays for phosphorus-solubilizing (both inorganic and organic phosphate) and cellulose-hydrolyzing activities were performed [94, 95]. Colonies exhibiting clear zones indicated phosphate-solubilizing or cellulose-hydrolyzing activity. Selected bacterial isolates were subjected to in vitro colorimetric determination of IAA production using Salkowski’s reagent (2% of 0.5 M FeCl_3_ in 35% perchloric acid), following a standard protocol [96]. Appearance of a reddish to pinkish color in the solution indicated the presence of IAA in the cultured medium. All the assays were conducted in triplicates and repeated at least twice.

Antagonistic capacity of selected *Pantoea* and *Xanthomonas* strains against *Magnaporthe oryzae* on potato dextrose agar (PDA) was performed as follows: fresh mycelial disks (diameter, 5 mm) of the fungi were inoculated onto the center of fresh PDA plates (diameter, 90 mm), and 5 μL of each saturated bacterial culture was dotted around the inocula at a distance of 30 mm. The plates were incubated at 25 °C for 3–4 days, and the antibiosis ability was determined by measuring the antagonistic zones. To test the antagonistic capacity for phytobacteria, 5 mL of *X. oryzae* pv. *oryzae* cultures was premixed with 50 °C Luria-Bertani medium and poured onto plates. Five microliters of each saturated strain culture was placed at the plate center and incubated at 28 °C for 2–3 days. Clear inhibition zones indicated antagonistic activity. All the assays were independently performed at least twice in triplicate.

## Supplementary Information

**Additional file 1: Supplementary Figure 1.** Sample collection and physicochemical properties. a, Samples were collected from four regions. SY, Sanya; LF, Langfang; NC, Nanchang; XB, Xishuangbanna. n represents the sample numbers. b, Physicochemical properties of bulk soil samples from four regions. Means with the same letters are not statistically different based on a T-test (*p* < 0.05). c, Redundancy analysis based on Bray-Curtis dissimilarity method of bulk soil ASV table and physicochemical properties from four regions. RDA1 and RDA2 show the first and second components of the RDA analysis, respectively. Regions are highlighted by ellipse and point shape. Significance of microbial community dissimilarities among different groups are based on a permutation test (*p* = 0.005).

**Additional file 2: Supplementary Figure 2.** Average Good’s coverage and rarefaction curves of six microhabitats. a, Bulk, bulk soil; b, Rhizo, rhizosphere; c, Root, root endosphere; d, Stem, stem endosphere; e, Seed-P, parental seed endosphere; f, Seed, offspring seed endosphere. Good’s coverage estimates are calculated in mothur based on 10,000 iterations. Rarefaction curves are generated showing the number of ASVs using 100 steps as rarefaction calculations, relative to the number of total sequences. The dashed vertical line indicates the number of sequences normalized from each sample to calculate alpha diversity.

**Additional file 3: Supplementary Figure 3.** ASV level alpha diversity of rice-associated bacterial microbiomes based on compartment and breed variation.

**Additional file 4: Supplementary Figure 4.** Taxa sorted by sampling areas from different compartments.

**Additional file 5: Supplementary Figure 5.** Z-P plot of ASVs on compartment variation. The within-module connectivity (*Zi*) and among-module connectivity (*Pi*) of each node were calculated to classify putative keystone ASVs in the network. Node topologies were organized into four categories: module hubs (highly connected nodes within modules, *Zi* > 2.5), network hubs (highly connected nodes within the entire network, *Zi* >2.5 and *Pi* > 0.62), connectors (nodes that connect modules, *Pi*> 0.62), and peripherals (nodes connected within modules with few outside connections, *Zi* < 2.5 and *Pi* < 0.62.

**Additional file 6: Supplementary Figure 6.** The occurrence frequency of core ASVs in different compartments. Each replicate was considered as a single group, and the sequencing/read number > 1 was classified as presented.

**Additional file 7: Supplementary Figure 7.** Average Nucleotide Identity based on BLAST for a selection of bacterial genomes. a, *Pantoea*: 21 strains and 6 type strains. b, *Xanthomonas*: 27 strains and 3 type strains). The prefix character “P_” of the strain name in the horizontal label indicates that the strain is of parental origin. The prefix character “O_” of the strain name in the vertical axis label indicates that the strain is derived from offspring. Cell colors indicate similarity scaled from low (blue) to high (red). ANIb values were calculated using fastANI.

**Additional file 8: Supplementary Figure 8.** Pan-genome analysis of *Pantoea* (*n* = 21) and 27 *Xanthomonas* (*n* = 27) strains. a and c, Flower plots indicating the core and pan genes in isolated *Pantoea* and *Xanthomonas* strains. The numbers of pan or core genes between subsets of genome were shown. b and d, Pan-genome accumulation curves. The curve of the pan-genome plot and the heaps law model (alpha = 0.21 for *Pantoea* and 0.19 for *Xanthomonas*) indicated an open-pan genome among these isolates.

**Additional file 9: Supplementary Figure 9.** Functional characterization of the core genome of *Pantoea* and *Xanthomonas* strains. a and b, COG annotation of core genome from *Pantoea* and *Xanthomonas* strains. c and d, KEGG annotation of core genome from *Pantoea* and *Xanthomonas* strains. Colors in panels a and c correspond to those depicted in panel b and d.

**Additional file 10: Supplementary Table 1.** Total number of reads and read length before and after quality checking and normalization.

**Additional file 11: Supplementary Table 2.** Assigned numbers of bacterial phyla, genera and ASV under different locations, microhabitats and varieties.

**Additional file 12: Supplementary Table 3.** Alpha diversity indicated by Chao1 and Shannon under microhabitats, locations and varieties variations with related P and F values.

**Additional file 13: Supplementary Table 4.** Taxonomic composition of the bacterial communities under different plant microhabitats, locations at the class or genus level.

**Additional file 14: Supplementary Table 5.** Topological properties of networks for bacterial ASVs under different microhabitats.

**Additional file 15: Supplementary Table 6.** Hub nodes revealed by ASV's *Zi* and *Pi* based on microhabitat variation.

**Additional file 16: Supplementary Table 7.** Detailed taxonomy classification of core rice endophytic microbiota with 70% occurrence in the collected samples.

**Additional file 17: Supplementary Table 8.** Genomic characteristics of *Pantoea* and *Xanthomonas* isolates.

**Additional file 18: Supplementary Table 9.** Biocontrol and plant growth-promoting properties of selected *Pantoea* and *Xanthomonas*.

## Data Availability

Scripts for bioinformatic analysis and plotting used in this study are available at https://github.com/lagrangemyn/RiceMicrobiome. Raw sequence data for *16S rRNA* gene amplicons are available on the NCBI Sequence Read Archive (SRA), accession number: PRJNA837349. Whole genome data have been deposited in DDBJ/ENA/GenBank under the accession number PRJNA843221. Draft genome data have been deposited in DDBJ/ENA/GenBank under the accession number PRJNA844595.
