# Supplementary figures and images for: Dynamics of rice microbiomes reveal core vertically transmitted seed endophytes
Source: Microbiome. 2022 Dec 9;10:216. doi: 10.1186/s40168-022-01422-9 (PMC9733015; doi:10.1186/s40168-022-01422-9)

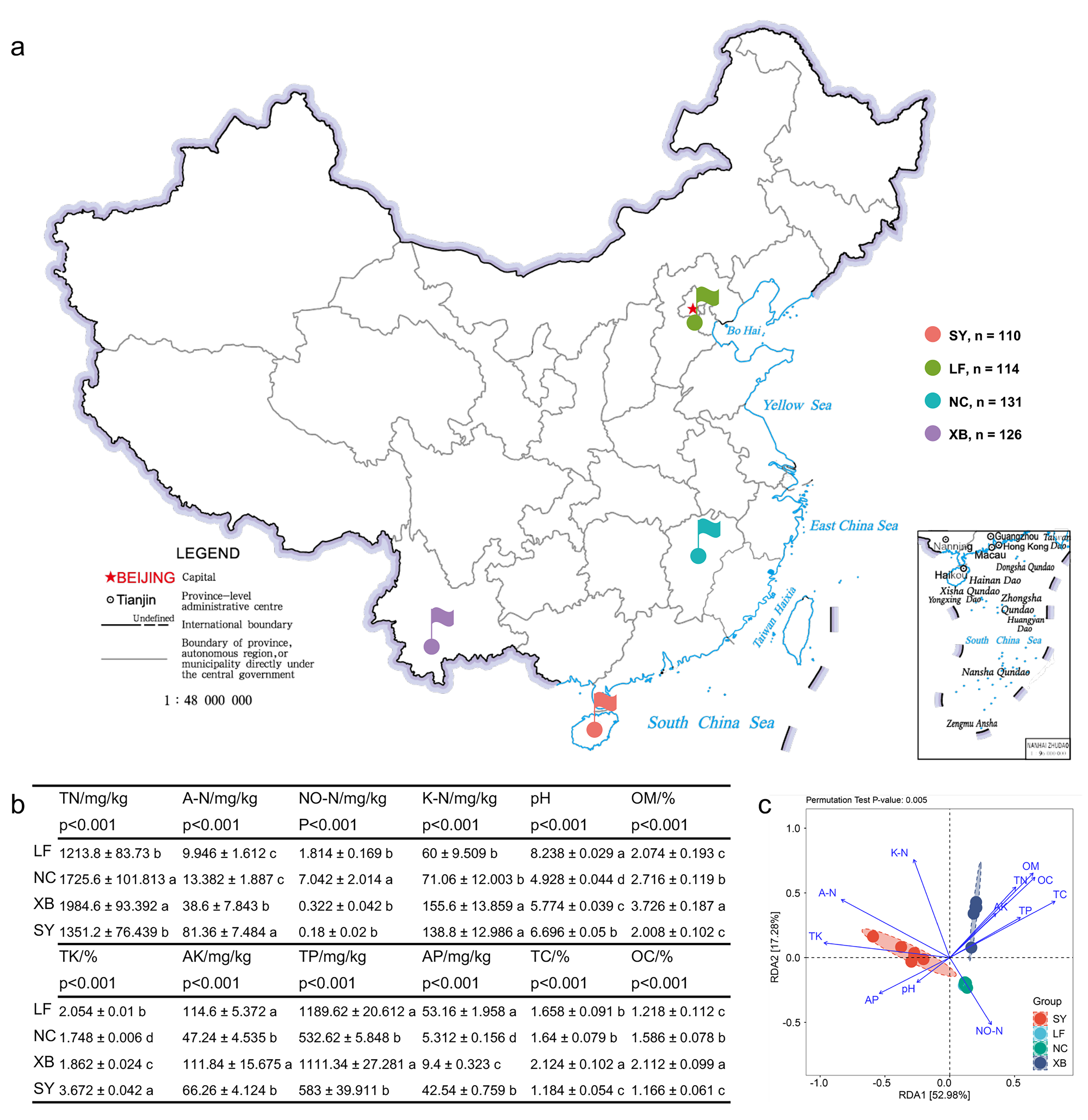

Supplement: Supplementary file 2 — Additional file 1: Supplementary Figure 1. Sample collection and physicochemical properties. a, Samples were collected from four regions. SY, Sanya; LF, Langfang; NC, Nanchang; XB, Xishuangbanna. n represents the sample numbers. b, Physicochemical properties of bulk soil samples from four regions. Means with the same letters are not statistically different based on a T-test (p < 0.05). c, Redundancy analysis based on Bray-Curtis dissimilarity method of bulk soil ASV table and physicochemical properties from four regions. RDA1 and RDA2 show the first and second components of the RDA analysis, respectively. Regions are highlighted by ellipse and point shape. Significance of microbial community dissimilarities among different groups are based on a permutation test (p = 0.005). [file 40168_2022_1422_MOESM1_ESM.tif]

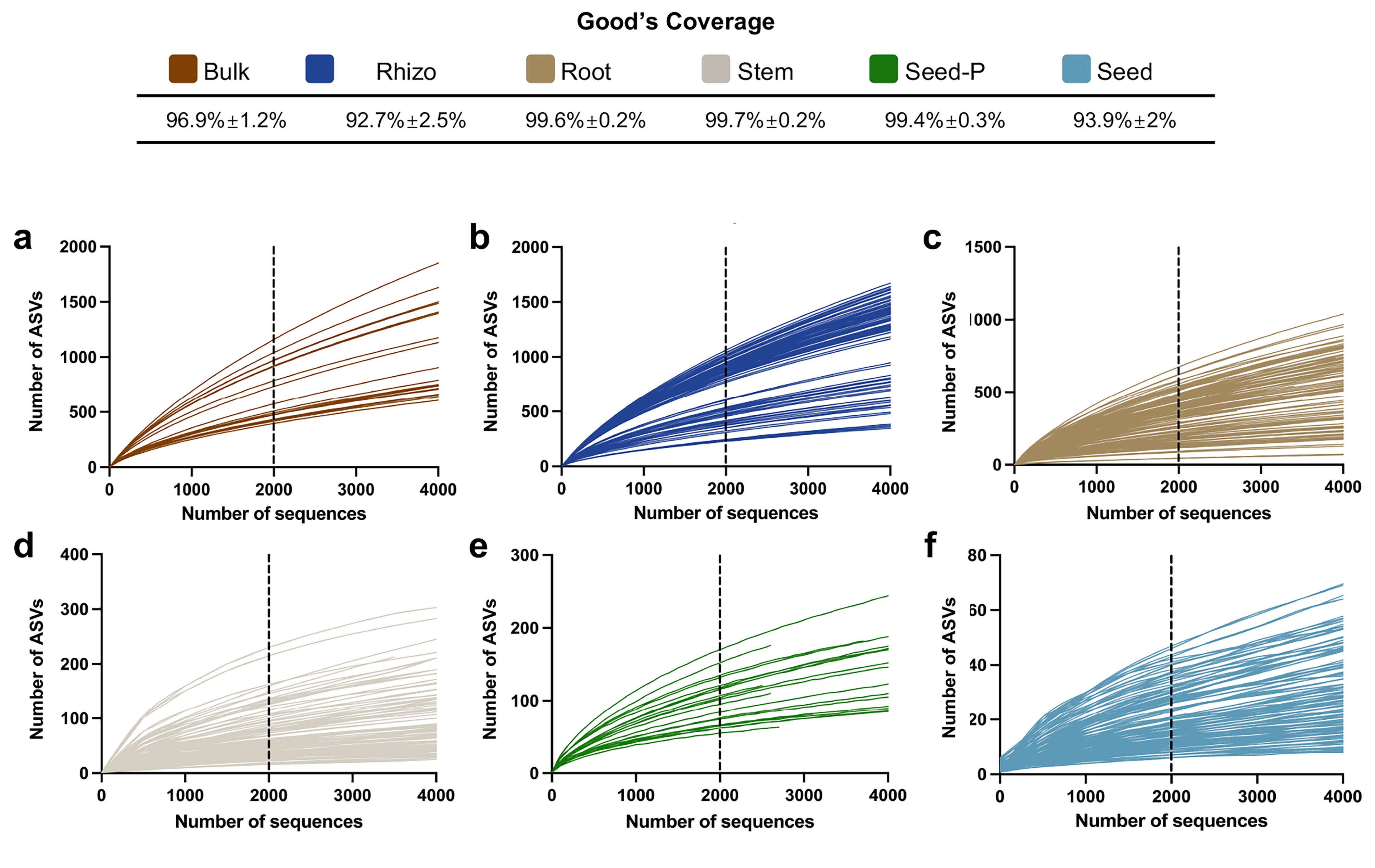

Supplement: Supplementary file 3 — Additional file 2: Supplementary Figure 2. Average Good’s coverage and rarefaction curves of six microhabitats. a, Bulk, bulk soil; b, Rhizo, rhizosphere; c, Root, root endosphere; d, Stem, stem endosphere; e, Seed-P, parental seed endosphere; f, Seed, offspring seed endosphere. Good’s coverage estimates are calculated in mothur based on 10,000 iterations. Rarefaction curves are generated showing the number of ASVs using 100 steps as rarefaction calculations, relative to the number of total sequences. The dashed vertical line indicates the number of sequences normalized from each sample to calculate alpha diversity. [file 40168_2022_1422_MOESM2_ESM.tif]

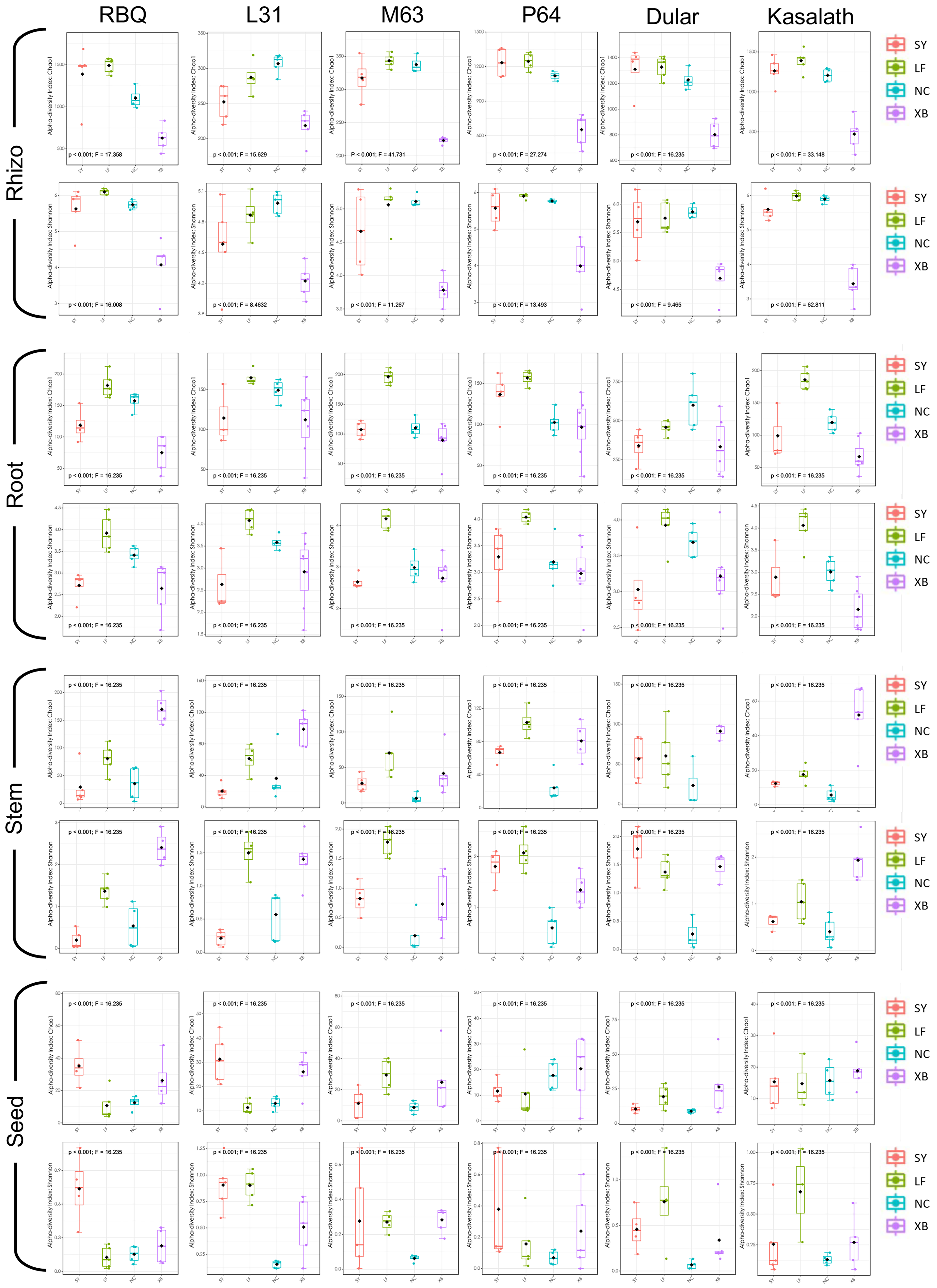

Supplement: Supplementary file 4 — Additional file 3: Supplementary Figure 3. ASV level alpha diversity of rice-associated bacterial microbiomes based on compartment and breed variation. [file 40168_2022_1422_MOESM3_ESM.tif]

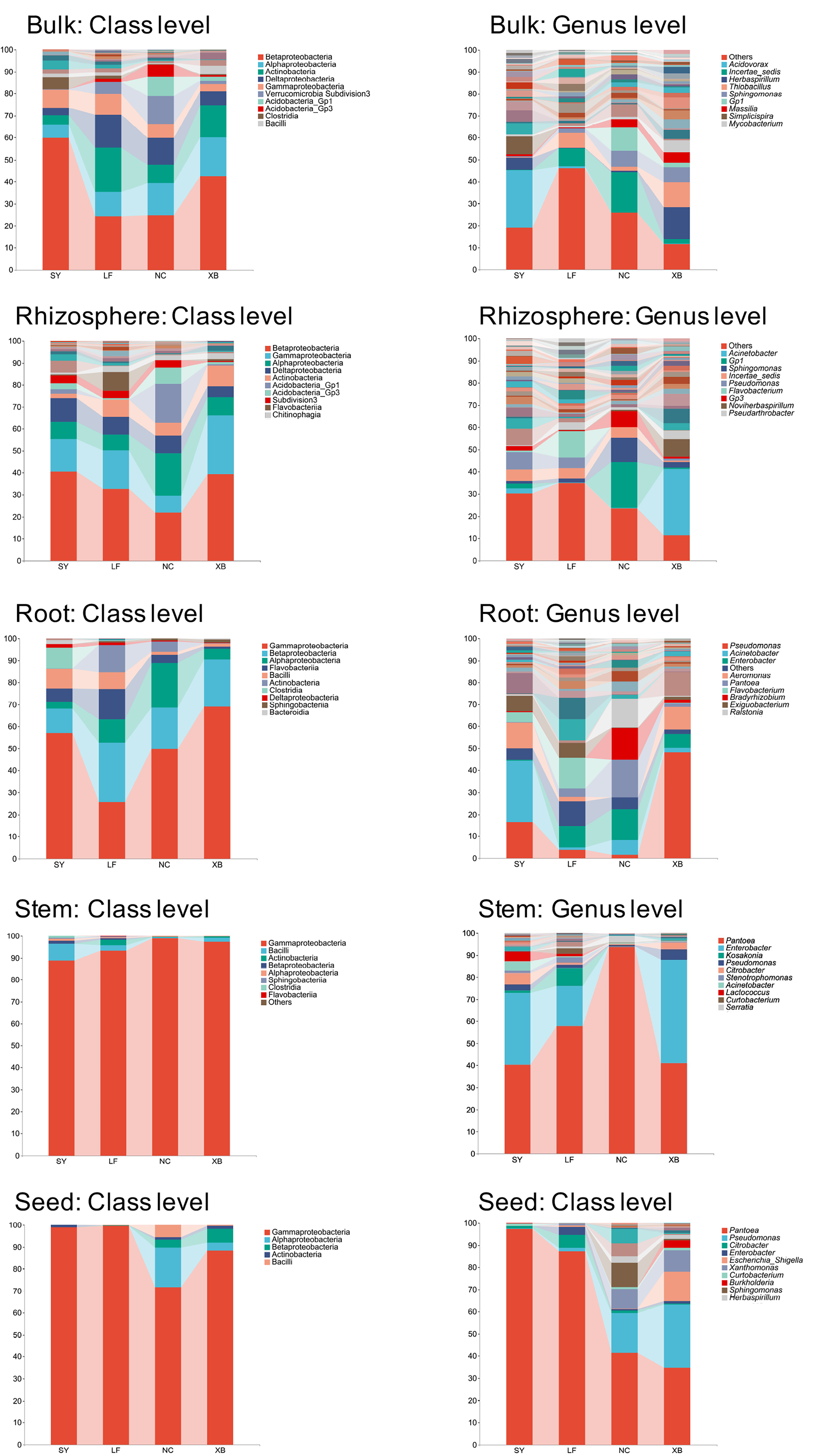

Supplement: Supplementary file 5 — Additional file 4: Supplementary Figure 4. Taxa sorted by sampling areas from different compartments. [file 40168_2022_1422_MOESM4_ESM.tif]

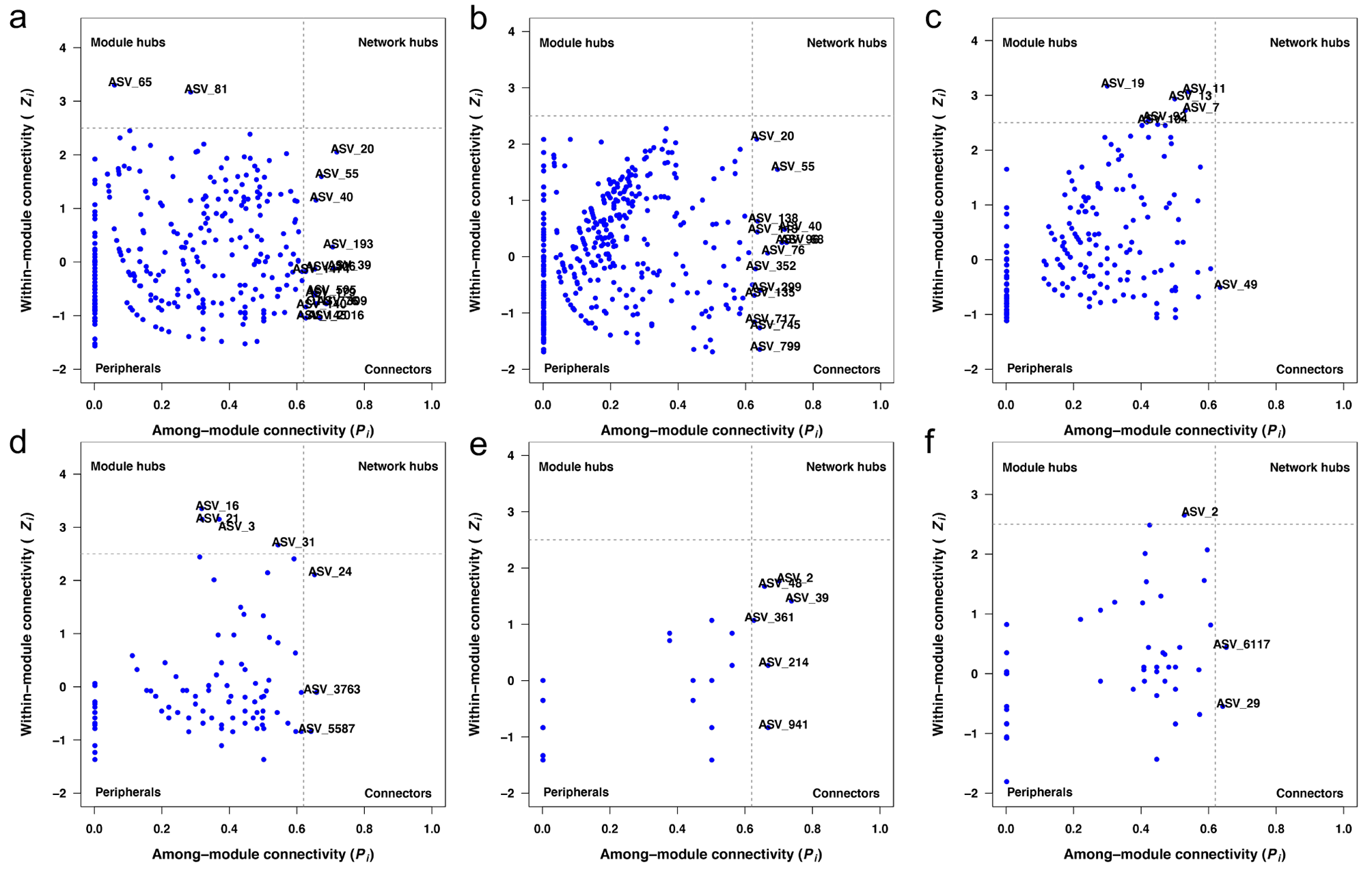

Supplement: Supplementary file 6 — Additional file 5: Supplementary Figure 5. Z-P plot of ASVs on compartment variation. The within-module connectivity (Zi) and among-module connectivity (Pi) of each node were calculated to classify putative keystone ASVs in the network. Node topologies were organized into four categories: module hubs (highly connected nodes within modules, Zi > 2.5), network hubs (highly connected nodes within the entire network, Zi >2.5 and Pi > 0.62), connectors (nodes that connect modules, Pi> 0.62), and peripherals (nodes connected within modules with few outside connections, Zi < 2.5 and Pi < 0.62. [file 40168_2022_1422_MOESM5_ESM.tif]

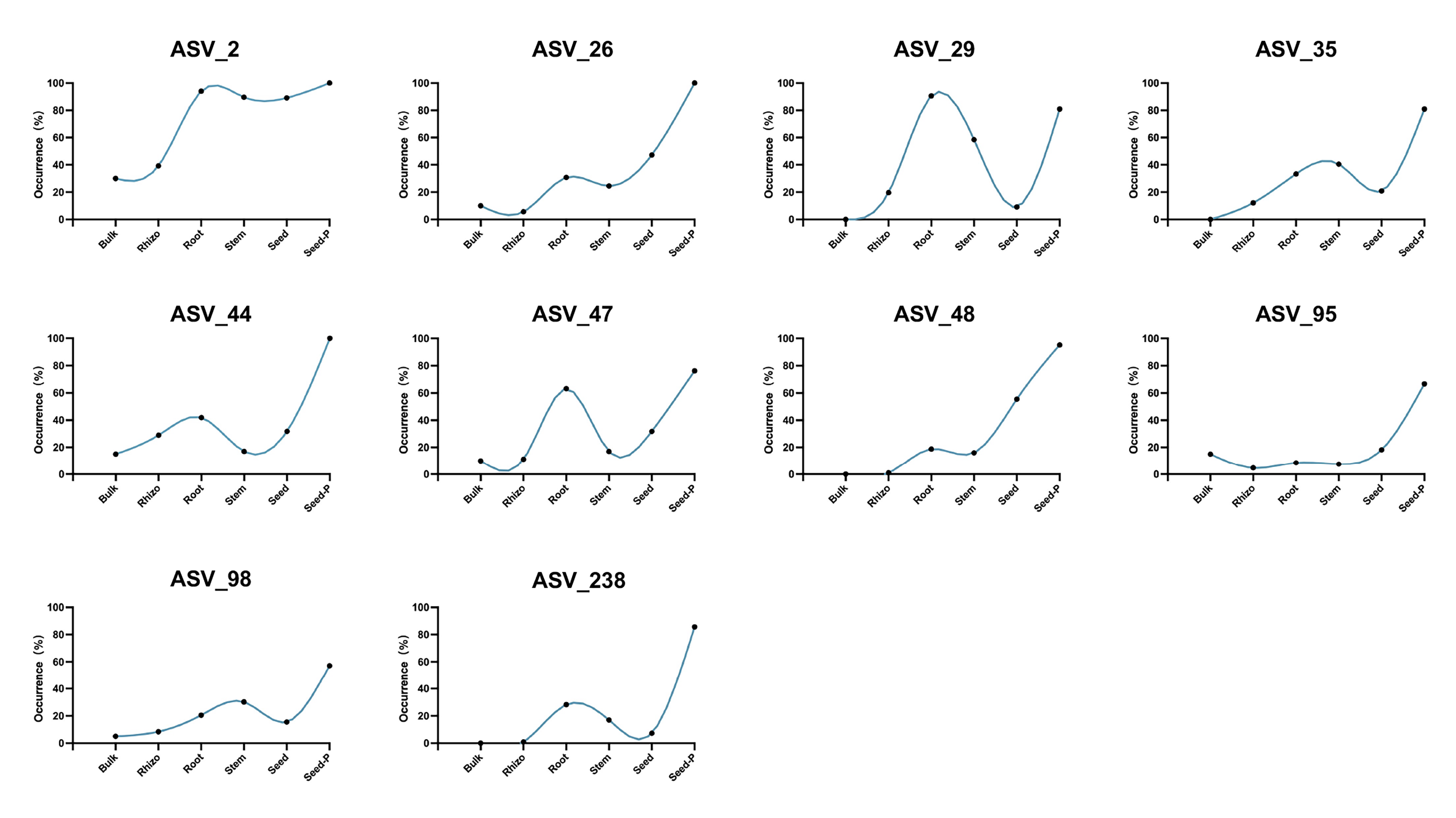

Supplement: Supplementary file 7 — Additional file 6: Supplementary Figure 6. The occurrence frequency of core ASVs in different compartments. Each replicate was considered as a single group, and the sequencing/read number > 1 was classified as presented. [file 40168_2022_1422_MOESM6_ESM.tif]

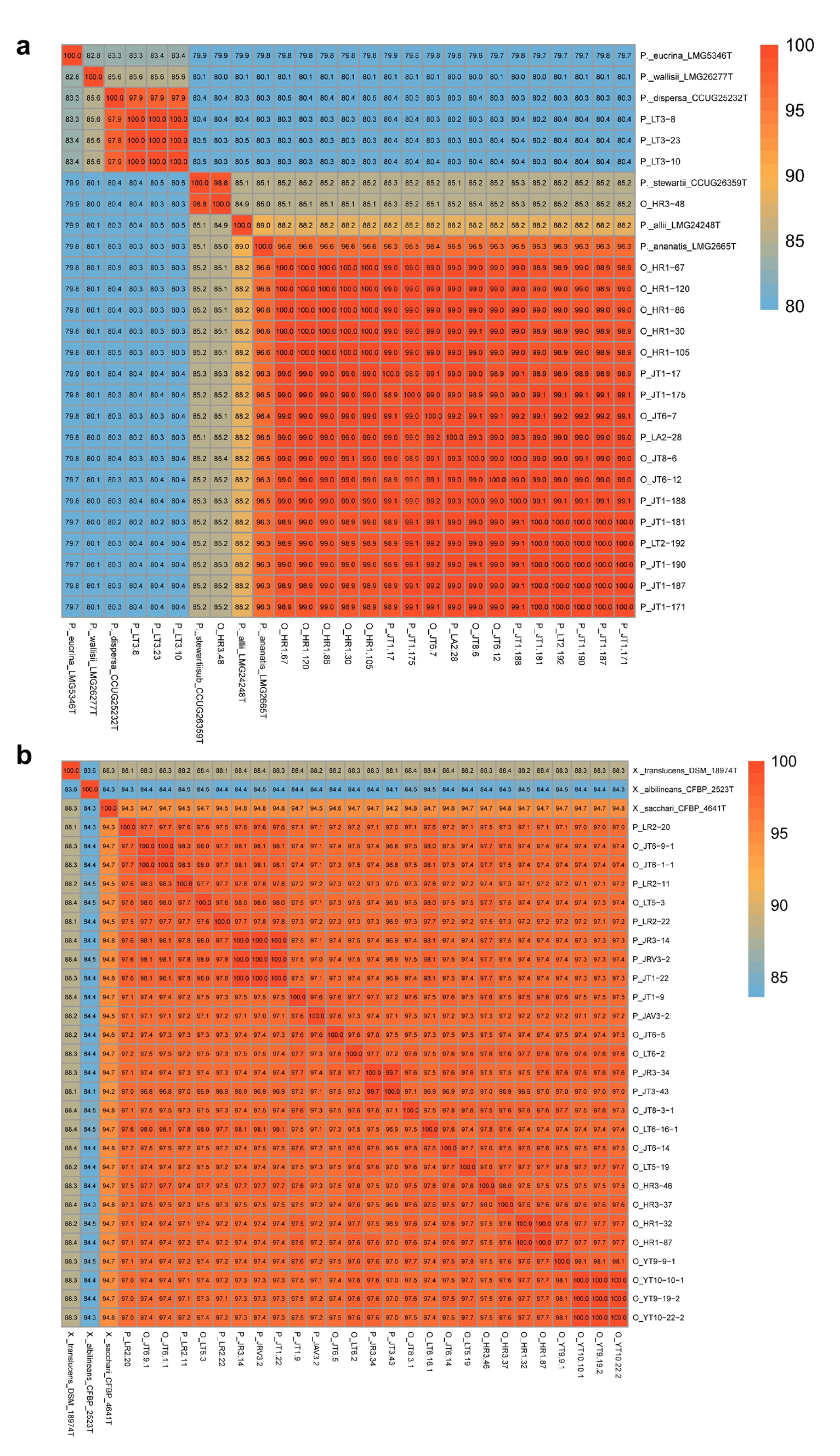

Supplement: Supplementary file 8 — Additional file 7: Supplementary Figure 7. Average Nucleotide Identity based on BLAST for a selection of bacterial genomes. a, Pantoea: 21 strains and 6 type strains. b, Xanthomonas: 27 strains and 3 type strains). The prefix character “P_” of the strain name in the horizontal label indicates that the strain is of parental origin. The prefix character “O_” of the strain name in the vertical axis label indicates that the strain is derived from offspring. Cell colors indicate similarity scaled from low (blue) to high (red). ANIb values were calculated using fastANI. [file 40168_2022_1422_MOESM7_ESM.tif]

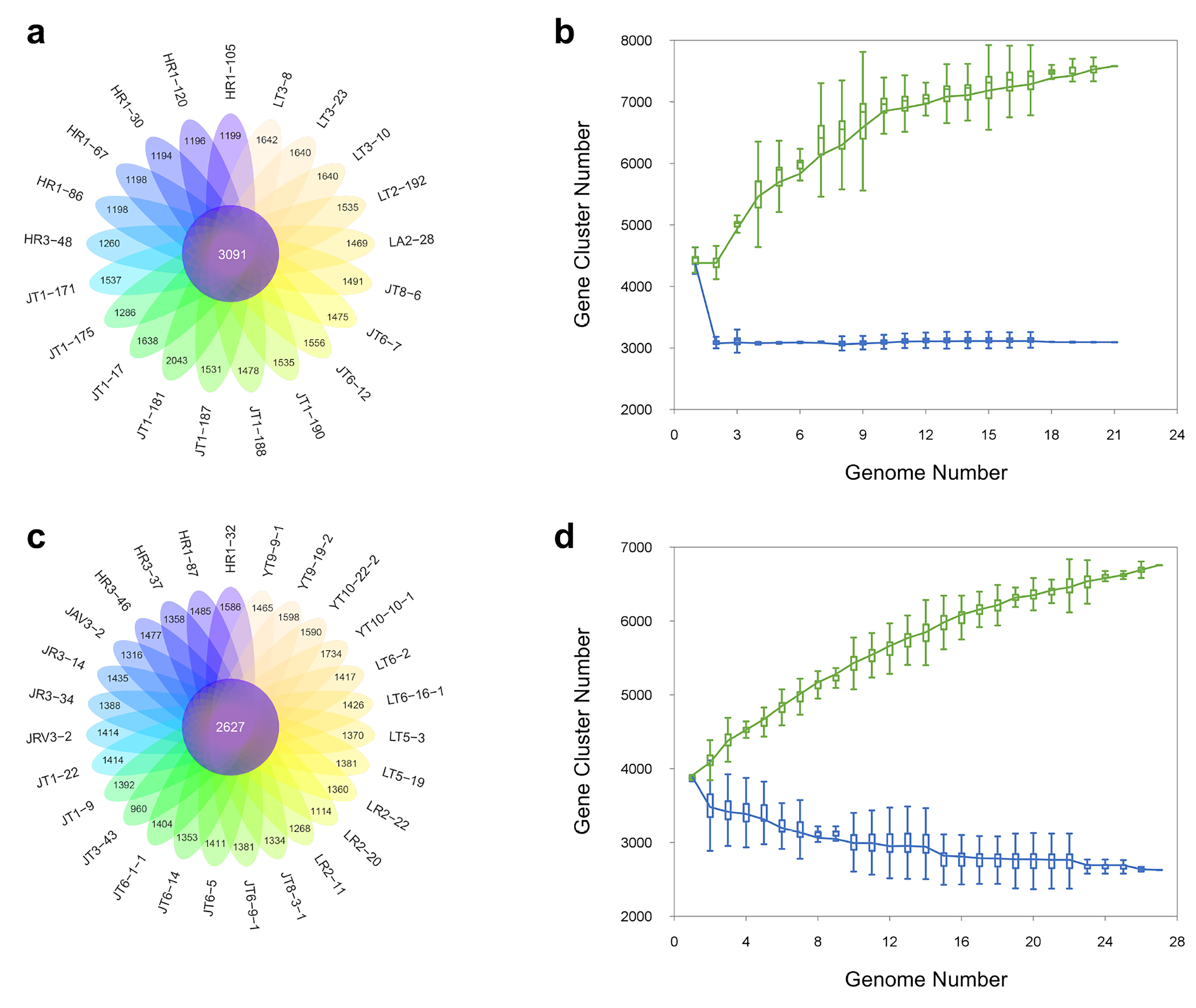

Supplement: Supplementary file 9 — Additional file 8: Supplementary Figure 8. Pan-genome analysis of Pantoea (n = 21) and 27 Xanthomonas (n = 27) strains. a and c, Flower plots indicating the core and pan genes in isolated Pantoea and Xanthomonas strains. The numbers of pan or core genes between subsets of genome were shown. b and d, Pan-genome accumulation curves. The curve of the pan-genome plot and the heaps law model (alpha = 0.21 for Pantoea and 0.19 for Xanthomonas) indicated an open-pan genome among these isolates. [file 40168_2022_1422_MOESM8_ESM.tif]

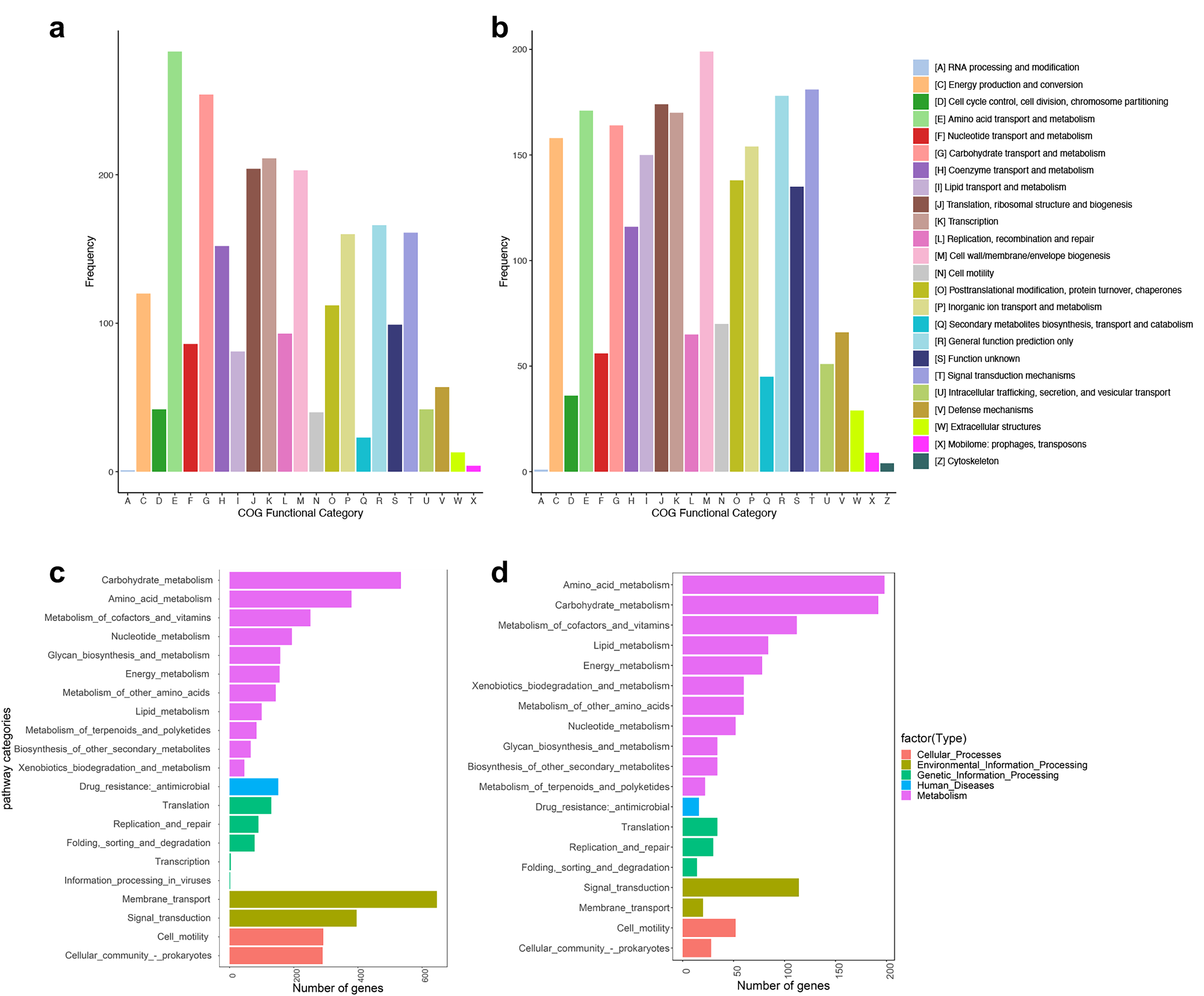

Supplement: Supplementary file 10 — Additional file 9: Supplementary Figure 9. Functional characterization of the core genome of Pantoea and Xanthomonas strains. a and b, COG annotation of core genome from Pantoea and Xanthomonas strains. c and d, KEGG annotation of core genome from Pantoea and Xanthomonas strains. Colors in panels a and c correspond to those depicted in panel b and d. [file 40168_2022_1422_MOESM9_ESM.tif]
